# Mapping Coeliac Toxic Motifs in the Prolamin Seed Storage Proteins of Barley, Rye, and Oats Using a Curated Sequence Database

**DOI:** 10.3389/fnut.2020.00087

**Published:** 2020-07-17

**Authors:** Matthew Daly, Sophie N. Bromilow, Chiara Nitride, Peter R. Shewry, Lee A. Gethings, E. N. Clare Mills

**Affiliations:** ^1^Division of Infection, Immunity and Respiratory Medicine, Faculty of Biology, Medicine and Health, Manchester Institute of Biotechnology, University of Manchester, Manchester, United Kingdom; ^2^Centre for Crop Genetic Improvement, Rothamsted Research, Harpenden, United Kingdom; ^3^Waters Corporation, Wilmslow, United Kingdom

**Keywords:** gluten, sequence database, barley, rye, oats, coeliac disease, wheat

## Abstract

Wheat gluten, and related prolamin proteins in rye, barley and oats cause the immune-mediated gluten intolerance syndrome, coeliac disease. Foods labelled as gluten-free which can be safely consumed by coeliac patients, must not contain gluten above a level of 20 mg/Kg. Current immunoassay methods for detection of gluten can give conflicting results and may underestimate levels of gluten in foods. Mass spectrometry methods have great potential as an orthogonal method, but require curated protein sequence databases to support method development. The GluPro database has been updated to include avenin-like sequences from bread wheat (*n* = 685; GluPro v1.1) and genes from the sequenced wheat genome (*n* = 699; GluPro v 1.2) and *Triticum turgidum* ssp durum (*n* = 210; GluPro v 2.1). Companion databases have been developed for prolamin sequences from barley (*n* = 64; GluPro v 3.0), rye (*n* = 41; GluPro v 4.0), and oats (*n* = 27; GluPro v 5.0) and combined to provide a complete cereal prolamin database, GluPro v 6.1 comprising 1,041 sequences. Analysis of the coeliac toxic motifs in the curated sequences showed that they were absent from the minor avenin-like proteins in bread and durum wheat and barley, unlike the related avenin proteins from oats. A comparison of prolamin proteins from the different cereal species also showed α- and γ-gliadins in bread and durum wheat, and the sulphur poor prolamins in all cereals had the highest density of coeliac toxic motifs. Analysis of ion-mobility mass spectrometry data for bread wheat (cvs Chinese Spring and Hereward) showed an increased number of identifications when using the GluPro v1.0, 1.1 and 1.2 databases compared to the limited number of verified sequences bread wheat sequences in reviewed UniProt. This family of databases will provide a basis for proteomic profiling of gluten proteins from all the gluten containing cereals and support identification of specific peptide markers for use in development of new methods for gluten quantitation based on coeliac toxic motifs found in all relevant cereal species.

## Introduction

Wheat is one of the most important crop globally, with the combined production with related cereal species (barley (*Hordeum vulgare*), rye (*Secale cereale*), and oats (A*vena sativa*) exceeding ~95,026 million tonnes in 2017 (1). The major storage protein fractions in cereal grains are defined as prolamins based on their solubility in mixtures of alcohol and water and their high contents of glutamine and proline. These proteins account for up to 80% of total protein content in wheat, barley and rye (2, 3) but are relatively minor components in oats (4). The gluten proteins of wheat form a visco-elastic network when wheat flour is mixed with water, which enables the production of leavened bread and other products (including pasta and noodles). Although these properties are not shared by the prolamins in related cereals (barley, rye and oats), restricting the use of these cereals in food processing, their sequences are related to those of wheat gluten proteins. Consequently, although the term gluten strictly applies only to wheat prolamins, it is defined in a regulatory context as; “the protein fraction from wheat, barley, rye, oats or their crossbred varieties and derivatives thereof, to which some persons are intolerant and that is insoluble in water and 0.5 M NaCl” (5).

Cereal seed storage prolamins can be distinguished based on their solubility in aqueous alcohol mixtures as either alcohol-soluble monomeric prolamins or alcohol-insoluble polymeric glutenins (6, 7). The monomeric prolamins can be further classified into α-,γ-, and ω-types based on their electrophoretic mobility whilst the components of the polymeric fractions, can be classified after reduction as belonging to either high molecular weight (HMW) and low molecular weight (LMW) groups (8). The prolamins from different cereal species are termed as either gliadins (wheat), hordeins (barley), or secalins (rye). A further group, originally identified in oats, are called avenins and have previously been classified either into three groups termed α-, β-, and γ-avenins according to electrophoretic mobility at low pH (9) or into eleven groups termed Avn-1-1 to Avn-10 based elution profiles from ion-exchange chromatography followed by RP-HPLC (10). In addition, molecular approaches have been used to classify them into A-, B-, and C-avenins, based on their repetitive domain structure (11). Subsequently sequences encoding proteins related to oat avenins have been identified in bread wheat (12), *T. turgidum* ssp durum (13) and barley (14). Based on sequence homology these have been called “avenin-like” proteins, and have been classified in wheat as being either a or b type avenins, with different subtypes indicated by Arabic numerals (12); it has also been proposed that the avenin-like protiens from wheat be termed farinins (15). They have also been shown to have a positive effect on dough strength in bread wheat (16) as well as pathogen resistance (17). The prolamin seed storage proteins are also important because of their ability to elicit both IgE- and non-IgE immune mediated adverse reactions in some individuals. Coeliac disease is a non-IgE immune-mediated food intolerance, affecting ~1% of the global population (18) and is triggered by prolamin seed storage proteins present in some cereal grains; wheat, barley, rye and, in some patient populations, oats (18, 19). Ingestion of dietary gluten leads to a variety of symptoms in susceptible individuals such as diarrhoea, abdominal distension, villous atrophy and an increased risk of adenocarcinoma and lymphoma (20). As a consequence of their high contents of proline, these prolamin seed storage proteins are partially resistant to gastric, pancreatic and brush border proteases resulting in longer peptide fragments reaching the small intestinal mucosa. Following the action of tissue transglutaminase (tTG) in the gut epithelium, which deamidates glutamine residues, some of these digestion-resistant fragments contain nine amino acid residue motifs capable of binding to certain variants of the Human Leukocyte Antigen class II receptors, HLA-DQ2 and HLA-DQ8. In addition to stimulating the production of antibodies to both tTG and gluten, the peptides activate gluten-specific naïve CD4^+^ T cells leading to an inflammatory response that causes the gut mucosa to flatten, reducing its absorptive capacity. These T cell epitopes have been termed coeliac toxic motifs (21, 22). Although the number of coeliac toxic motifs in a protein fragment can be correlated to its immunotoxicity, there are many other factors involved. These include resistance to gastrointestinal digestion, how effective peptides are as substrates for tTG as well as the binding affinity for HLA and capacity to activate T cells. Indeed, there is correlation between the likelihood of a sequence being deamidated by tTG and its ability to activate T cells in individuals with coeliac disease (23, 24). By contrast IgE-mediated food allergies have been associated with sensitisation to particular cereal storage prolamins including wheat-dependent exercise-induced anaphylaxis (WDEIA) a condition associated with sensitisation to ω5-gliadins (also known as Tri a 19). Sensitisation to other seed storage has been described including α- and γ- gliadins, LMW and HMW subunits of glutenin [Tri a 20, 21, 26, and 36; (25, 26)] together with non-gluten proteins, notably the non-specific lipid transfer protein (LTP; Tri a 14).

No cures exist for either coeliac disease, or IgE-mediated food allergies, and the only treatment is strict avoidance of gluten or wheat-containing foods. In order to help patients with coeliac disease avoid gluten the CODEX Alimentarius Commission developed recommendations for gluten-free foods which has been implemented in regulations across the world (27). In the EU, if cereal-derived food ingredients (such as wheat starch or dextrin) contain <20 mg/Kg they can be labelled as gluten-free, although wheat must still be declared on the ingredient label (28, 29). The available validated methods for gluten quantification are immuno-based assays, which suffer from several limitations and can lead to false detection and quantification. The high sequence homology between prolamins in cereal species can cause partial reactivity of the antibodies to wheat, barley, rye and oats, and the potential reactivity with contaminating wild grass species. Moreover, incomplete extraction of proteins and the use of incorrect conversion factors can further compound these issues (30–33).

An alternative to immunoassays is mass spectrometry, which has been used as an orthogonal method of quantifying gluten in complex matrices (34–37). However, accurate identification of proteins using mass spectrometry-based proteomics approaches relies heavily on the quality of the protein database or annotated genome against which the mass spectra are searched. Various databases are available such as UniProt containing both reviewed (Swiss-Prot) and unreviewed (TrEMBL) protein sequences (38), and the NCBI Protein Database (39). Although curated and draft genomes are available for some plant species, including wheat, barley and rye (40–42). These are inevitably cultivar specific, can be incomplete and often contain partial sequences. Furthermore, the reviewed UniProtKB/SwissProt database contains only 56 prolamin sequences combined from bread wheat, *Triticum turgidum* ssp durum, barley, rye, and oats. Some of these originate from protein sequencing and are not complete protein sequences [e.g., UniProt sequence accession Q09095; (43)]. In order to reduce redundancy in the database UniProtKB/Swiss-Prot the protein produced from a single gene at a species level, is provided as a single entry choosing a canonical sequence based on at set of criteria, one of which is sequence length, with isoforms being provided as alternative sequences under the main entry (44, 45). This curation process means that the number of prolamin sequences in reviewed UniProt has reduced from 61 (accessed 14.5.2019) to 56 accessed 5.12.2019).

An alternative is to create custom databases combining reported protein sequences from other databases such as NCBI and EST sequences in order to facilitate proteomic analysis, although these are not all publicly available (34). One publicly available curated prolamin sequence database is ProPepper, a tool containing ~2,480 cereal prolamin sequences data (46) although the sequences are not available in a format suitable for direct mining of mass spectrometry data. Other repositories are of curated sequences implicated in IgE-mediated allergies (47) and include the IUIS allergen nomenclature database which seeks to curate well-defined allergen sequences and has 40 sequences from wheat, barley and rye, although they include both inhalant and food allergens (48). Another curated allergen sequence database is AllergenOnline, which contains 2,129 peer-reviewed sequences (49). Such allergen sequence databases are of limited usefulness in searching mass spectrometric data since they are not comprehensive for a given organism and can use conflicting nomenclature. For example, the allergen Tri a 20 is referred to a γ-gliadin in the IUIS database which includes two accessions, but a further six sequence accessions are classified as Tri a 20 in AllergenOnline ver 19.

In order to address the need for a curated sequence database to facilitate analysis of proteomic data, the GluPro database was created containing 630 discrete unique full length bread wheat prolamin protein sequences encompassing both the gliadin and glutenin fraction and applied to characterisation of the bread wheat prolamin proteome (50). However, it does not include the avenin-like sequences from bread wheat and sequences from the wheat genome (cv Chinese Spring) which limits its utility. The sequence database has now been enlarged with avenin sequences to give GluPro v1.1 and further enriched with wheat genome sequences to give GluPro v 1.2. In addition the informatics pipeline developed by Bromilow and co-workers (50) has been applied to develop curated prolamin sequences from other cereal species including pasta wheat (*Triticum turgidum* ssp durum; GluPro v 2.1), barley (GluPro v 3.0), rye (GluPro v 4.0), and oats (GluPro v 5.0). These sequence sets were then compiled into a compendium database of gluten proteins from different cereal species (GluPro v 6.1). The resulting curated sequences were then analysed to determine the distribution of known coeliac toxic motifs using the AllergenOnline Celiac Disease (CD) Novel Protein Risk Assessment Tool (http://www.allergenonline.org/celiachome.shtml) (49). The expanded GluPro v 6.1 database will enable discovery proteomics data to be mined more effectively, in order to identify effective peptide markers. These are required for development of targeted, quantitative mass spectrometry methods for determination of gluten in food, which may originate from bread wheat, *T. turgidum* ssp durum, barley, rye and oats.

## Materials and Methods

### Methods

#### Database Construction

Sequence sets of seed storage prolamins from *T. turgidum* ssp durum (GluPro v 2.0), *H. vulgare* (GluPro v 3.0), *S. cereale* (GluPro v 4.0) and *A. sativa* (GluPro v 5.0) were created independently and an update of the bread wheat (*T. aestivum*) database was undertaken to enrich it with avenin-like sequences (GluPro v 1.1) (Figures S1, S2).

In stage I the entire UniProt (accessed 04.01.2019 for GluPro v 3.0, 4.0 and 5.0, and 29.07.2019 for GluPro v 2.0) and NCBI Protein (accessed 12.02.2019 for GluPro v 3.0, 4.0 and 5.0, and 29.07.2019 for GluPro v 2.0) databases were mined using the search terms; “prolamin,” “gluten,” “gliadin,” “glutenin,” “hordein,” “secalin,” or “avenin” using the origin species set to either “*Triticum turgidum* ssp durum,” “*Hordeum vulgare,”* “*Secale cereale,”* or “*Avena sativa*.” When populating the GluPro v 1.1 sequence set, the search term was “avenin” and the origin species was set to “*Triticum aestivum.”* In each case, all sequences were downloaded in FASTA format and combined into origin species-specific sequence sets. Redundant sequences were removed using the DB Toolkit software (51) with UniProt accessions being preferentially retained. Partial, non-seed storage prolamins and sequences containing ambiguous amino acids were then removed from the databases manually (sequence set one) if they lacked homology to reviewed seed storage prolamin sequences (8, 52). This was done, as although the sequence may have some protein level evidence, identifying these proteins experimentally using shotgun proteomics would not be possible. “X” denotes ambiguous amino acids in protein sequences; they arise due to either the presence of multiple sequences showing different amino acids, or poor quality data that is unable to distinguish between amino acids (53, 54).

In Stage II the curated sequence sets for each cereal species were then separately searched against the entire UniProt database (01.03.2019 for GluPro v 3.0, 4.0 and 5.0, and 19.11.2019 for GluPro v 2.0) using protein-protein BLAST (Basic Local Alignment Search Tool). Based on a minimum sequence homology of ~30% the first 250 sequences were downloaded regardless of origin species. This was below the 40% threshold Addou et al. (55) suggested for inferring homology and was chosen to ensure that all homologous proteins were recovered from searching which were then manually curated (see below). The sequences curated in Stage I and II were combined and subjected to another round of curation removing duplicates and partial sequences (Figure S1) to give databases for bread wheat (GluPro v 1.1), *T. turgidum* ssp durum (GluPro v 2.0), barley (GluPro v 3.0), rye (GluPro v 4.0), and oats (GluPro v 5.0). The species-specific sequence databases were then combined to give a complete seed storage prolamin sequence database (GluPro v 6).

In Stage III the recently published reference genome for *T. aestivum* cv. Chinese Spring (41) and draft genome available for *T. turgidum* ssp durum cv. Svevo (42), were then mined for further prolamin seed storage protein sequences (Figure S2). This was not necessary for barley as its draft genome (cv. Morex) is available as a reference proteome on UniProt and sequences from this translated genome were downloaded during creation and curation of GluPro v 3.0. Translated genomes of *T. aestivum* and *T. turgidum* ssp durum were downloaded from Ensembl Plants in FASTA format yielding 133,346 and 196,105 peptide/protein sequences, respectively, for each species. These files were then converted to BLAST databases using the standalone BLAST+ software (56) and the entire GluPro v 6 BLAST searched against them using Genome Workbench v 3.1.0 (57) with an Expect value of 10. After further manual curation (as described for Stages I and II) novel sequences were added to the respective species-specific database to give GluPro v 1.2 and GluPro v 2.1, respectively. These were then added to GluPro v 6 to give GluPro v 6.1. Although a draft genome is available for *S. cereale* cv. Lo7, it is unavailable for download in a translated format (58). However, a BLAST server of the transciptome is available at http://webblast.ipk-gatersleben.de/ryeselect/ (accessed 12.11.2019). Therefore, the GluPro v 6.0 database was BLAST searched against this transcriptome using an Expect value of 10, and homologous sequences were retrieved and manually curated. Where possible, transcript identifiers were replaced with UniProt accession numbers.

#### Sequence Alignment and Analysis

Sequences were aligned using Clustal Omega (59) and resulting alignments downloaded in Multiple Sequence File (MSF) format and visualised in Jalview (60). A phylogenetic tree was created in Jalview based on average distance (a type of unweighted pair group method with arithmetic mean) and BLOSUM62, viewed and edited in FigTree (v1.4.3). Phylogenetic tree building was undertaken using average distance rather than approaches such as neighbour-joining, as an equal rate of evolution was assumed i.e., a molecular clock. This analysis was only used to cluster proteins into their respective protein groups and not to determine evolutionary origin. Resulting sequence classifications were manually cross-referenced based on available literature regarding N-terminal sequence, mass, repeat sequence and phylogeny (8, 11, 61, 62). Sequences classified as being within the same protein group from the same species were subject to multiple pairwise alignments such that every sequence was compared to every other sequence and average percentage homology calculated (Tables S1–S4). Master sequences with protein level evidence were identified where possible for each protein group from each species that represented that protein roup.

#### Mapping of Coeliac Toxic Motifs

Sequences present in the databases were further analysed with regard to the distribution of coeliac toxic motifs using the online database AllergenOnline (49) that contained 1,013 coeliac toxic peptide sequences at the time of analysis (11.03.2019 and 02.12.2019). It should be noted that some of these peptides are fragments of others and therefore not unique. Using the “Exact Peptide Match” function all 1,013 peptides available were mapped against the full sequences from the curated databases. From this function three measurements were taken: number of unique coeliac toxic motifs per sequence, density of unique coeliac toxic motifs and sequence coverage by coeliac toxic motifs as a percentage of total sequence length. The number of unique coeliac toxic motifs was simply the number of motifs that were present in the sequence, although this excluded instances where unique motifs occurred more than once in the sequence and is irrespective of that fact that some motifs are fragments of others. The density of unique coeliac toxic motifs was calculated by taking the number of unique coeliac toxic motifs present in the sequence and dividing by the sequence length. Sequence coverage by coeliac toxic motifs was calculated using Protein Coverage Summarizer software (v1.3.6794) where all 1,013 sequences in the AllergenOnline CD Tool were mapped against the sequences. This calculation ignores the fact that some sequences present in the AllergenOnline CD Tool are fragments of each other.

#### Mass Spectrometry Analysis

Seed from *T. aestivum* (cultivars Chinese Spring and Hereward) were obtained from Rothamsted Research (Harpenden, UK), two grains crushed separately and proteins extracted with 50 mM Tris HCl (pH 8.8), 50 mM DTT and 0.02% (w/v) RapiGest™ at 60°C with sonication and vortexing every 5 min (50). Extracts were clarified by centrifugation for 10 min at 10,000 × g, supernatants removed and then further reduced, alkylated with iodoacetamide and digested with chymotrypsin as previously described (50). Resulting peptides were desalted and concentrated using C18 ZipTips (Waters Corporation, Wilmslow, UK). Peptides were subsequently analysed using liquid chromatography ion mobility mass spectrometry (LC-IM-MS-MS). For the chromatography the mobile phases of solvent A consisted [0.1% (v/v) formic acid/99.9% (v/v) water] and solvent B consisted [0.1% (v/v) formic acid/99.9% (v/v) acetonitrile]. Chromatographic separation was undertaken using a linear gradient (flow rate 300 nL/min) from 3 to 40% (v/v) solvent B over 90 min using a M-class ACQUITY UPLC system (Waters Corporation) equipped with a NanoEase 1.8 μm HSS T3 C18 (75 μm × 150 mm) column (Waters Corporation) attached to a SYNAPT G2-Si QTOF mass spectrometer (Waters Corporation). Data were acquired using a data independent approach in positive ion mode over the mass range m/z 50–2,000 with a 0.5 s spectral acquisition time and one cycle of low and elevated energy data was acquired every 1 s (50).

#### Analysis of Mass Spectrometric Data

IM-MS-MS data were processed using Progenesis QI for Proteomics (v 3) using the Ion Accounting workflow. After ion detection, low- and high-energy mass events are time-aligned to precursor-product ion tables, and then filtered to remove any precursor ions under 750 Da and all product ions under 350 Da. A searchable database is then selected and a reversed decoy database is appended, and the algorithm completes a pre-search step where, using Bayesian inference, model parameters are adjusted and fine-tuned. The algorithm then completes several passes of database searching to match theoretical peptides to observed mass events. This iterative process of peptide spectrum matching can improve the number of peptides identified from IM-MS-MS compared to other mass spectrometry database search programs such as Mascot and ProteinLynxGlobalSERVER (63). Once imported, sequence sets were searched against the GluPro v 1, 1.1, and 1.2 databases, and reviewed prolamin sequences from *T. aestivum* downloaded from UniProt (downloaded 20.01.2019). Cleavage was set to chymotrypsin with cleavage occurring at Y, W, F or L unless followed by a P with up to two missed cleavages. False discovery rate (FDR) was set to 1% and mass tolerance for peptide and fragment ions were set to 10 and 20 ppm, respectively. The distribution of q-values obtained for all analyses is shown in Figure S7 with only identifications with q values ≤ 0.01 being considered. Apex 3D parameters were set to 150 counts for low energy intensity threshold and 30 counts for high energy. Carbidomethylation of cysteine was selected as a fixed modification, whereas oxidation of methionine, hydroxylation of proline, deamidation of glutamine or asparagine and N-terminal pyroglutamatic acid were all selected as variable modifications. Protein identifications were only considered valid if at least one unique peptide was identified for that protein in at least 2/3 technical replicates in both biological replicates, and with a peptide score >5.

## Results

### Database Construction and Sequence Classification

Initially the GluPro v 1.0 database was enriched with avenin-like sequences from *T. aestivum*. A total of 11,917 sequences were downloaded from both UniProt and NCBI Protein databases. Additional prolamin seed storage protein sequence databases were also developed for other cereal species including *T. turgidum* ssp durum, barley, rye and oats (Figures S1, S2). The majority of these sequences were duplicates as the different search terms may return the same protein. For example, the protein with UniProt accession P06470 was returned when searched for “gliadin,” “glutenin,” and “hordein” and was therefore downloaded three times. As such, all databases were reduced to ~1% of the original size once duplicates had been removed. These included sequences with the same accession number and the same sequence with different accession numbers that have been deposited in the UniProt and NCBI databases more than once. BLAST searching of sequences identified eight avenin-like sequences from bread wheat, 10 sequences from *T. turgidum* ssp durum (two HMW glutenin subunits, four LMW glutenin subunits and four α-gliadin sequences), two C hordeins from barley, and two ω-secalins from rye. Once mis-assigned sequences, partial sequences and sequences with ambiguous amino acids were removed the databases comprised 182 sequences (*T. turgidum* ssp durum; GluPro v 2.0; Table S1), 64 (barley; GluPro v 3.0; Table S2), 41 (rye; GluPro v 4.0; Table S3) and 27 sequences (oats; GluPro v 5.0; Table S4), respectively. Fifty-five sequences attributed to avenin-like proteins from *T. aestivum* were added to the original Glu Pro v 1.0 prolamin sequence set (*T. aestivum*; GluPro v 1.1). These were combined to give a more complete “cereals containing gluten” database comprising 998 sequences (Glu Pro v 6).

Mining of the Chinese Spring wheat genome yielded 14 new sequences; nine α-gliadins, three further avenin-like sequences and two δ-gliadin sequences recently reported by Altenbach et al. (64). Interestingly no HMW glutenin subunit sequences were present in the translated genome. Interrogation of the cDNA database indicated five HMW glutenin subunit sequences (Ax, Bx, Dx, By, and Dy), however these were annotated as non-translating CDS and as such did not appear in the translated genome. Four sequences contained “N” in the sequence indicating an unknown nucleotide, and one sequence encoded a protein only 340 amino acids in length. Three HMW glutenin subunit sequences were identified from another sequenced genome using *T. aestivum* cv Chinese Spring (65). Twenty-eight sequences were also added by mining the *T. turgidum* ssp durum translated genome including α-gliadin, avenin-like and low molecular weight glutenin subunit protein sequences. These were added to the bread wheat and *T. turgidum* ssp durum databases GluPro 1.1 and GluPro 2.0 databases to create GluPro 1.2 and 2.1, respectively (Figure S2). These were then combined with GluPro v 3.0-5.0 to create GluPro v 6.1 containing 1041 sequences, an increase of 4.2%. It was not necessary to mine the *H. vulgare* cv. Morex translated genome, as it is already available as a reference proteome in UniProt, eighteen sequences in the barley database GluPro 3.0 having originated from the sequenced genome. The *S. cereale* translated genome was also mined but no sequences were identified that were not already present in the rye database GluPro v 4.0. UniProt accession numbers, evidence level and supporting literature for each sequence in the database are available in Supplementary Material (Tables S1–S4). In addition all the databases are available in FASTA format from https://figshare.com/search?q=10.6084%2Fm9.figshare.12613154.

### Phylogenetic Analysis of Prolamin Sequences From Cereals Containing Gluten

**Figure 1:**
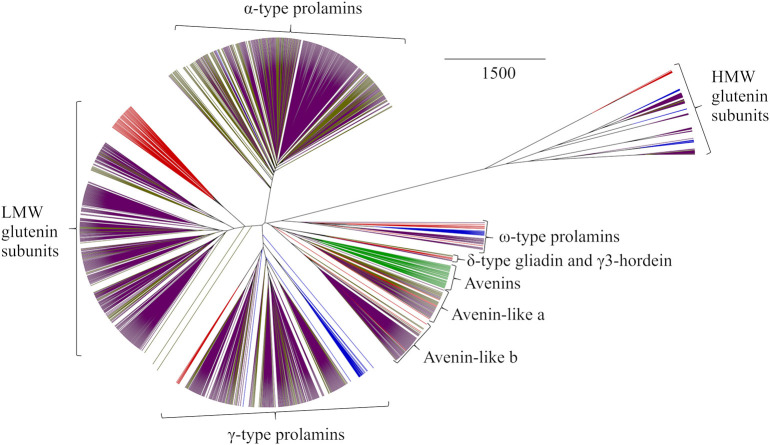
Average distance phylogenetic tree of immature sequences from *T. aestivum* (purple), *T. turgidum* ssp durum (gold), *H. vulgare* (red), *S. cereale* (blue), and *A. sativa* (green). The scale bar indicates the number of amino acid substitutions per site.

Phylogenetic analysis of all sequences in GluPro v 6.1 revealed clustering into the expected protein groups between and within species similar to that observed previously for the original *T. aestivum* GluPro v 1.0 (50) (Figure 1). Briefly, proteins separated into seven groups; the sulphur-rich α-type prolamins, Low Molecular Weight (LMW) glutenin subunits, γ-type prolamins, avenin-like a, b and avenins, δ-type prolamins with γ3-hordeins, the sulphur-poor ω-type prolamins and finally the High Molecular Weight (HMW) glutenin subunits.

The α-type prolamins are only present in bread wheat and *T. turgidum* ssp durum and therefore form a distinct branch on the phylogenetic tree with a single α-type prolamin sequence from rye being identified. This sequence was reported based on a cDNA sequence (66) and may be wrongly assigned or derived from Triticale since rye does not contain α-prolamin genes. Triticale (also called Triticosecale) is derived from hybridization of wheat and rye and therefore contain α-prolamins encoded by the Triticum genome (67). The phylogenetic analysis also revealed the known similarity of the polymeric LMW glutenin subunit types with the monomeric gliadin-like α- and γ-prolamins. LMW glutenin subunits from *T. turgidum* ssp durum clustered largely with sequences from bread wheat, the wheat sequences falling into seven groups which had characteristic N-terminal sequences including into the more phylogenetically distant LMW-i group; the B1 and B3 hordeins from barley also clustered alongside the LMW subunits of glutenin (50).

The sulphur-poor ω-type prolamins were more distantly related, the polymeric HMW subunits of glutenin being the most distantly related type of prolamin sequence. HMW glutenin subunits separate based on length, and in the case of bread wheat, the variation was linked to the chromosomal locations of the encoding genes. The HMW secalins and HMW glutenin subunits from *T. turgidum* ssp durum were less divergent then those from bread wheat. The lower level of variation in HMW subunits sequences in *T. turgidum* ssp durum and rye may relate to the fact that these species are tetraploid and diploid, respectively, whereas, bread wheat is hexaploid. However, the limited variation observed may simply be because fewer sequences were available from rye and *T. turgidum* ssp durum. The D-hordeins were more closely related to the y-type HMW glutenin subunits present in wheat and rye than to the x-type subunits of wheat. Three other types of prolamin were also identified using the phylogenetic analysis which clustered together with the avenins of oats and the avenin-like proteins in bread wheat*, T. turgidum* ssp durum, and barley. The avenins from oats all clustered on one branch with the avenin-like proteins from other cereal species falling into two other clusters corresponding to the avenin-like a and b groups previously identified in wheat (12). Phylogenetic analysis also allowed identification of the recently discovered δ-type prolamin present in bread wheat (64, 68, 69), and now also identified in *T. turgidum* ssp durum. Interestingly, these sequences clustered with the three γ3-hordein sequences from barley, and appear related to the avenins and avenin-like proteins, demonstrating the homologous nature of these proteins but further complicating nomenclature regarding the prolamins.

In order to interrogate the sequence relationships between the different types of prolamin, master sequences were identified for which protein level evidence existed (Table 1) and aligned C-terminal segments shown in Figure S3. Protein-level evidence was lacking for δ-gliadin and avenin-like proteins from *T. turgidum* ssp durum, certain avenin-like proteins from barley, α-type prolamin from rye and A-type avenin from oats. For these classes of prolamins candidate master sequences were selected with a proline plus glutamine content >30% to confirm they were prolamins and a high sequence homology to every other sequence in the protein group (Table 1). Within-protein group sequence homology between species was also high (>50%), further demonstrating the correct classification of these sequences. Extremely high homology (of 92.25 and 91.73%, respectively), was observed between the avenin-like a and b proteins from bread wheat, *T. turgidum* ssp durum and barley. This analysis also confirmed that, although avenins from oats were distinctly separated from the gliadins (Figure 1), that they are indeed prolamins, although their proline plus glutamine content is lower (32–42%) than other prolamin sequences (Table 1) (4). This is because the avenin proteins lack the long repetitive domains present in other prolamins, indicating that the coding genes could either be related to ancestral forms of seed storage genes that have since evolved a repetitive domain, or the result of a more recent evolution that have removed the repetitive domain (77). The avenin-like sequences of *T. turgidum* ssp durum typically had a content of proline plus glutamine ranging from 22 to 34%. This lower level is due to a subset of avenin-like a proteins having shorter sequences, together with point mutations and deletions in the short polyglutamine region. The characteristic conserved skeleton of eight cysteine residues of the prolamin superfamily is demonstrated in all sequences apart from the ω-type prolamins and the HMW glutenin subunits (Figure S3) (78). The ω-type prolamins contain no cysteine residues and consist mostly of repeat motifs, and HMW glutenin subunits contain a longer central domain of repeat motifs that disrupts the characteristic cysteine residue backbone.

**Table 1:** Sequence similarity within protein groups between species.

**Protein group**	**Origin species**	**UniProt accession number**	**Evidence level**	**Supporting literature reference**	**% Sequence similarity**	**Proline + glutamine (%)**
α-type prolamins	*T. aestivum*	X2KVH9	Protein	(70)	84.15	48.67
	*T. turgidum* ssp durum	D2X6C9	Protein	(71)		49.46
	*S. cereale*	H8Y0F9	Genome	(66)		50.00
δ-type prolamins	*T. aestivum*	A0A2U8JD37	Protein	(64)	89.64	45.54
	*T. turgidum* ssp durum	A0A446IHB0	Genome	Manual submission L. Milanese Sep 2017		37.31
γ-type prolamins	*T. aestivum*	K7X1R6	Protein	(70)	65.29	50.55
	*T. turgidum* ssp durum	Q6EEW5	Protein	(71)		43.10
	*H. vulgare*	M0XYT2	Protein	(72)		47.22
		P17990	Protein	(72)		44.76
		P80198	Protein	(70)		44.98
	*S. cereale*	E5KZQ5	Protein	(70)		42.73
		E5KZP9	Protein	(70)		61.09
Group I avenins	*T. aestivum*	P0CZ07	Protein	(73)	92.25	35.00
	*T. turgidum* ssp durum	182970^*^	Genome	(42)		32.60
	*H. vulgare*	F2EGD5	Protein	(74)		31.82
Group II avenins	*T. aestivum*	P0CZ05	Protein	(73)	91.73	34.83
	*T. turgidum* ssp durum	A0A446WXS7	Genome	Manual submission L. Milanese Sep 2017		35.71
	*H. vulgare*	A7XUQ7	Genome	(75)		34.59
Group III avenins	*A. sativa*	L0L8A4	cDNA	(11)	62.98	35.29
		P80356	Protein	(70)		41.79
		Q09114	Protein	(70)		41.76
LMW glutenin subunits	*T. aestivum*	B2Y2S3	Protein	(70)	75.98	50.86
	*T. turgidum* ssp durum	A0A2P1BXV0	Protein	(71)		50.15
	*H. vulgare*	P06470	Protein	(70)		49.64
		I6TEV5	Protein	(70)		51.20
ω-type prolamins	*T. aestivum*	Q402I5	Protein	(70)	53.87	72.86
	*H. vulgare*	A0A287EIM7	Protein	(70)		69.98
	*S. cereale*	C4NFN9	Protein	(70)		68.64
HMW glutenin subunits	*T. aestivum*	G3FLC7	Protein	(70)	69.31	49.68
		Q94IJ6	Protein	(70)		43.78
	*T. turgidum* ssp durum	Q8RVX0	Protein	(71)		45.99
		A0A0E4G9A4	Protein	1(76)		44.40
	*H. vulgare*	Q84LE9	Protein	(70)		36.68
	*S. cereale*	Q94IK8	Protein	(70)		45.38
		Q94IL2	Protein	(70)		48.16

Sequence similarity was calculated using pairwise alignment of master sequences, alongside UniProt accession number, protein group, origin species, evidence level with supporting literature reference and proline and glutamine percentage. Accession number indicated by

* *was retrieved from mining of the translated T. turgidum ssp durum genome (42)*.

**Figure 2:**
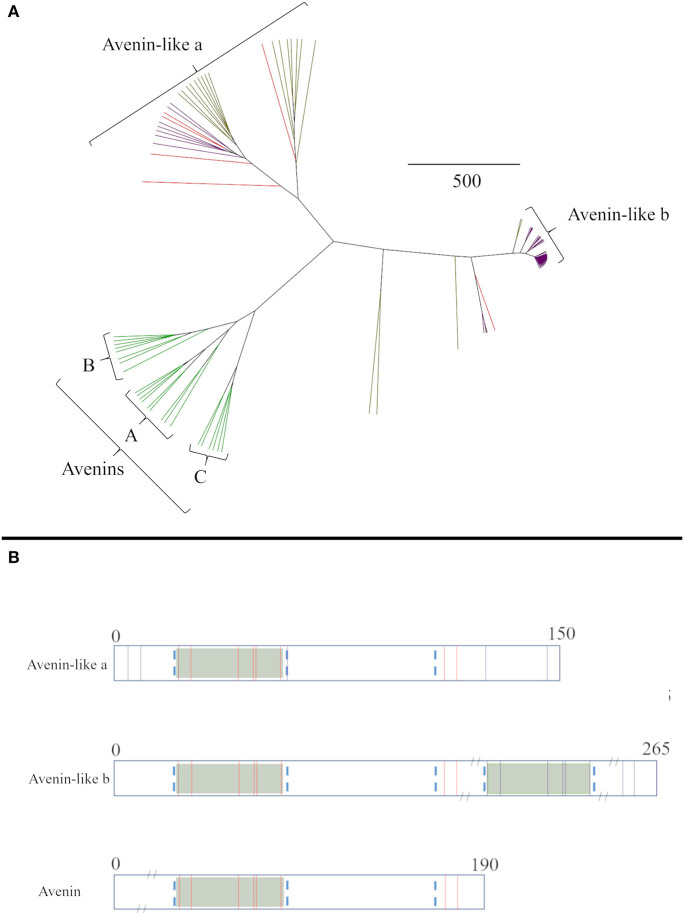
**(A)** Average distance phylogenetic tree of mature avenin and avenin-like sequences from *T. aestivum* (purple), *H. vulgare* (red) and *A. sativa* (green). The scale bar indicates the number of amino acid substitutions per site. **(B)** Schematic depiction including sequence length and position of cysteine residues present in Group I, Group II and Group III avenins. Conserved cysteine residues between all three groups are coloured red and non-conserved are shown in purple. Conserved domains that contain the characteristic prolamin cysteine residue skeleton are distinguished by green boxes and regions are outlined by blue dashed lines.

The relationships between the avenin-like proteins from the different cereal species were then analysed separately (Figure 2A). The avenin-like a proteins comprised sequences annotated as “avenin-like a” and “avenin-like” sequences from bread wheat and barley, respectively, and included “uncharacterised” protein sequences from *T. turgidum* ssp durum. The avenin-like b proteins, comprised protein sequences from bread wheat, *T. turgidum* ssp durum and barley that were annotated in Uniprot as being “avenin-like b” proteins. This separation results from differences in amino acid sequence, with “avenin-like b” sequences containing four to five more cysteine residues than “avenin-like a” due to duplication of a polyglutamine region containing the conserved prolamin cysteine residue skeleton (Figure 2B) (12, 77, 79). Avenins from oats clustered separately from sequences from the other cereal species (Figure 2A). Three avenin-like proteins from bread wheat, one from *T. turgidum* ssp durum and one from barley did not cluster with the other avenin-like proteins or avenins but formed separate branches, closer to the avenin-like b proteins. Interrogation of aligned amino acid sequences indicated high sequence homology between these sequences and avenin-like b sequences. However, a deletion of seven amino acids at residue number 209 and a further two amino acid insertion at position 242 was observed which explains the distance seen between the sequences on the phylogenetic tree.

**Figure 3:**
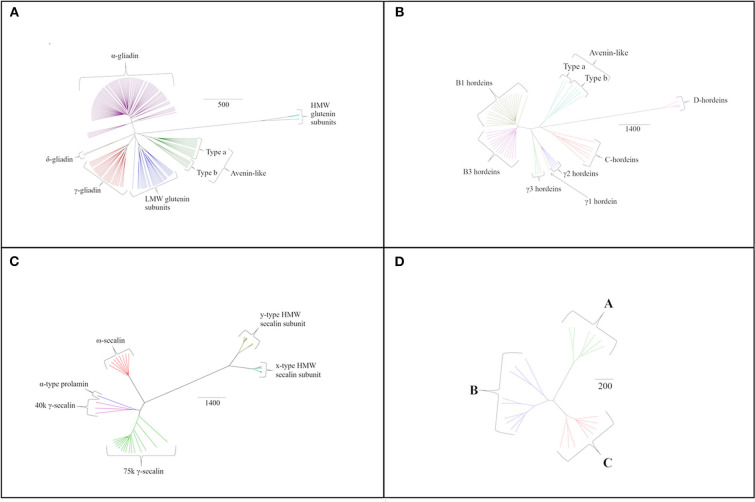
Species specific phylogenetic trees based on average distance and BLOSUM62 with different protein grouping for **(A)** *T. turgidum* ssp durum, **(B)** *H. vulgare* sequences, **(C)** *S. cereale* sequences, and **(D)** *A. sativa* sequences. The scale bar indicates the number of amino acid substitutions per site.

Individual species-specific phylogenetic trees provide further insights into the variations between the gluten components (Figure 3). Sequence homology was also determined within protein groups of the same species using all sequences available and is shown in Supplementary Material (Tables S1–S4). Similar to bread wheat, *T. turgidum* ssp durum prolamin sequences clustered into α-, δ- and γ-gliadins, low-molecular weight subunits of glutenin and avenin-like proteins together with the more distantly related HMW subunits of glutenin (Figure 3A). Interestingly, no ω-gliadins were identified in this organism despite the encoding regions being present on the short arm of all group 1 homoeologous chromosomes in bread wheat (80). Polypeptides with molecular weights consistent with ω-gliadins have been observed in electrophoretically separated extracts of *T. turgidum* ssp durum after immunoblotting with anti-ω5 gliadin antibodies (81). Peptide fragments of ω-gliadins have also been identified in simulated gastro-duodenal digests of pasta (82), although no sequences are available in either the UniProt or NCBI databases at present.

Analysis of the barley prolamin sequences allowed hordeins to be classified into avenin-like sequences, B1-, B3-, γ1-, γ2-, γ3- and C-hordeins together with the more distantly related D-hordeins (Figure 3B). Examination of the aligned γ-hordein sequences demonstrated that γ2-hordeins have extremely high homology to γ1-hordeins. Tanner et al. (83) suggested this is probably because γ2-hordeins are encoded by the γ1-hordein gene but have a post-translational deletion in the sequence. This results in proteins being expressed that are shorter by ~30 amino acids although evidence to support this suggestion is currently lacking.

Rye secalins could be classified into α-prolamins (the single sequence referred to above), ω-, 40 k γ-, 75 k γ-secalins and two types of HMW secalin subunit (Figure 3C). Differences in mass were used to separate the 40 k and 75 k γ-secalins, and the y-type and x-type HMW secalin subunits (x-type subunits being larger).

The avenins from oats could be further classified into A-, B-, or C-avenins based on their amino acid sequences (11) (Figure 3D and Table 2). Two B–avenins and one C-avenin classified by phylogenetic analysis contained repeat motifs that could place them either in group B or C (Figure 3 and Figure S4). An additional distinction can be made based on the number of cysteine residues: A-avenins contain nine cysteine residues and B- and C-avenins contain eight. A-avenins could therefore form intermolecular disulphide bonds due to the odd number of cysteine residues therefore making A-avenins polymeric (11). However, it should be noted that protein level evidence for the existence of A-avenins is currently lacking.

**Table 2:** Classification of avenin sequences from *Avena sativa* (Glu Pro 5.0 database).

**Phylogenetic classification**	**UniProt accession number**	**Shewry (62) classification**	**Repeat motif region I**	**Repeat motif region II**	**No. of cysteine residues**
Group A avenins	L0L6J7	Avenin-1-1, 1-2,−2,−4	PFM[Q_(1−5)_]	No repeat	9
	L0L5H3	pAv10 genomic clone			
	Q09071	pAV10 genomic clone			
	L0L8A0	Avenin-1-1, 1-2,−2,−4			
	L0L833	Avenin-1-1, 1-2,−2,−4			
	L0L837	Avenin-1-1, 1-2,−2,−4			
	L0L8A4	Avenin-1-1, 1-2,−2,−4			
	I4EP78	Avenin-1-1, 1-2,−2,−4			
	I4EP85	Avenin-1-1, 1-2,−2,−4			
	I4EP86	Avenin-1-1, 1-2,−2,−4			
Group B avenins	I4EP88	Avenin-1-1, 1-2,−2,−4	No repeat	VFQPQLQQ	8
	Q38794	AV45-X1 genomic clone	MLL[Q_(3−6)_]	FFQPQMQQ + VTQG	
	L0L4J1	Avenin-3			
	P80356	Avenin-3			
	L0L5I0	Avenin-3			
	L0L6J5	Avenin-3			
	Q2EPY2	Avenin-3			
	L0L4I8	Avenin-3		VFQPQLQQ	
	L0L6J0	Avenin-7/-8			
Group C avenins	L0L5H5	Avenin-5,−6.−7,−8,−9,−10	PFV[Q_(2−4)_]	FFQPQMQQ + VTQG	
	L0L5G8	Avenin-5,−6.−7,−8,−9,−10		VFQPQLQQ	
	Q09072	Avenin-5,−6.−7,−8,−9,−10			
	Q09114	Avenin-9			
	L0L8B6	Avenin-5,−6.−7,−8,−9,−10			
	L0L841	Avenin-5,−6.−7,−8,−9,−10			
	L0L6K5	Avenin-5,−6.−7,−8,−9,−10			
	L0L6K1	Avenin-5,−6.−7,−8,−9,−10			

*Avenin sequences from A. sativa UniProt accession number alongside previous classification according to Shewry (62) and the criteria used to classify them into either group A, B or C*.

### Analysis of Coeliac Toxic Motifs and IgE-Reactive Allergens

**Figure 4:**
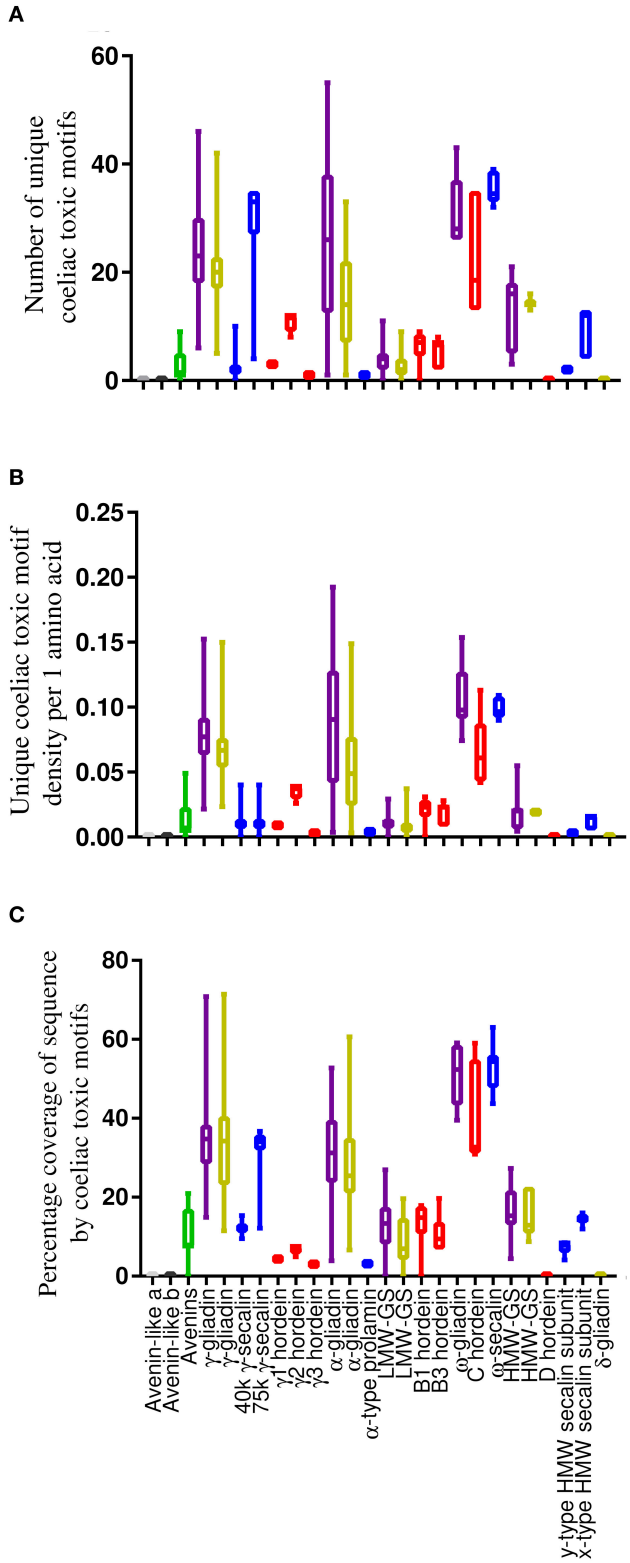
Analysis of sequences from the *T. turgidum* ssp durum, *H. vulgare, Secale cereale*, and *A. sativa* databases and avenin-like sequences from *T. aestivum* in the context of coeliac toxic motifs from the AllergenOnline database shown using box and whisper plots with sequences arranged according to structural classification. **(A)** shows the number of unique coeliac toxic motifs per sequence, **(B)** showing the density of motifs and **(C)** showing the sequence coverage by motifs calculated using the Protein Coverage Summarizer software. Bars coloured grey indicate sequences from *T. aestivum, T. turgidum* ssp durum, and *H. vulgare*, gold are sequences from *T. turgidum* ssp durum, red are sequences from *H. vulgare*, blue are sequences from *S. cereale* and green corresponds to sequences from *A. sativa*.

The average number of unique coeliac toxic motifs per sequence, coeliac toxic motif density and sequence coverage by coeliac toxic motifs was evaluated using the “exact peptide match” function from AllergenOnline and the repository of 1,013 coeliac toxic motifs contained within the database (Figure 4 and Table S5). There were large similarities between all metrics of coeliac toxic motif analysis within homologous protein groups across species although there were some differences compared to bread wheat (50). Thus, the S-poor prolamins in barley (C hordeins) and rye (ω-secalins) together with the rye 75 k γ-secalins and the α- and γ-gliadins from *T. turgidum* ssp durum generally carried the greatest number of coeliac toxic motifs across all the measures applied. Only the density of coeliac toxic motifs per residue varied, which was much lower for the rye 75 k γ-secalins. This is unlike bread wheat where α-gliadins contained the largest number and the highest density of coeliac toxic motifs (50) although it should be noted that this protein fraction is absent from barley and oats with only one unconfirmed sequence reported for rye. The avenins from oats contained a moderate load of coeliac toxic motifs and although 10.5% of A-avenins had no coeliac toxic motifs, the remainder carried at least one, as did the B- and C-type avenins. In contrast, no coeliac toxic motifs were identified in the avenin-like a and b proteins and δ-gliadins in any of the cereal species and were either low (e.g., the y-type HMW secalin subunit sequences) or absent (e.g., barley D hordeins) from the HMW subunits of glutenin.

The number of full length gluten protein sequences in Chinese Spring was recovered from the annotated genome sequence (64) supplemented with the total number of avenin-like sequences from the CS reference proteome available on UniProt (accessed16.01.2020). Databases used in searching were as follows; GluPro v 1 (n = 630), GluPro v 1.1 (n = 685) and GluPro v 1.2 (n = 699). Identifications were made using unique peptides of any length; those with unique peptides ≥5 amino acids in length are given in parentheses.

Analysis of IgE-reactive proteins, using the allergen sequences defined in the IUIS Allergen Nomenclature database (www.allergen.org) identified seven seed storage prolamin food allergens in bread wheat as follows: ω5-gliadin (Tri a 19; UniProt accession Q402I5), γ-gliadin (Tri a 20; UniProt accession A0A060N479 and Q9SYX8), α-gliadin (Tri a 21; UniProt accession D2T2K3), HMW GS Dx5 and Bx7 (Tri a 26; UniProt accession P10388 and Q45R38) and LMW GS GluB3-23 (Tri a 36; UniProt accession B2Y2Q7). *T. turgidum* ssp durum only contains one known allergenic protein, the non-specific lipid transfer protein (Tri tu 14; GenBank accession JF799976.1) Barley and rye only contain allergenic seed storage prolamin proteins located in the γ-type protein group; γ3-hordein (Hor v 20; UniProt accession P80198) and 75 k γ-secalin (Sec c 20; UniProt accession Q9S8B0 and Q9S8A7). The database contained no known allergenic proteins that mapped to oats.

### Application of the GluPro Bread Wheat Databases for Searching of Mass Spectrometry Data

The curated sequences from bread wheat (GluPro v 1.0, 1.1 and 1.2) were then used to analyse IM-MS-MS spectral libraries for bread wheat from cultivars Chinese Spring and previously published data from cv Hereward (50) and compared with searching against the reviewed UniProt sequences. Searching was undertaken using variable modifications for deamidation of glutamine and hydroxylation of proline, as these have previously been observed in plant proteomic data sets (84, 85). The distribution of q-values (adjusted *p*-values found using an optimised FDR approach) is shown in Figure S7 when mining the spectral libraries using the different databases. These density histograms show the distribution was as expected where the null features represent the flat portion whilst the “true” features all lie very close to zero. Since the FDR was set at 1% only proteins with a q value ≤ 0.01 were accepted as identifications. Example extracted ion chromatograms for selected peptides are shown in Figure S5. Using the UniProt reviewed prolamin sequences allowed a total of 16 and 19 proteins, respectively, in cvs Chinese Spring and Hereward to be identified (Table 3; Supplementary Datasheets 1, 2). In comparison searching using the curated gluten protein sequence databases yielded a much larger number of identifications, which were greater (40–42) for cv Hereward, compared to cv Chinese Spring (19–20). Modifying the searching databases to include the avenin sequences (GluPro v1.1) and the additional bread wheat accessions from the Chinese Spring Genome (GluPro v 1.2) had little impact on the total numbers of proteins identified but it did affect, in some cases, the numbers of proteins belonging to a specific class or the specific protein accessions identified. Thus, as expected, avenin-like proteins were identified using GluPro v1.1 and 1.2 although the numbers varied. Similarly the δ-gliadins were only identified using GluPro v 1.2, the database which actually contained these protein sequences as has previously been reported (64). Thus, using the curated sequence databases did offer an advantage over using a simple UniProt download.

**Table 3:** Summary of proteins identified by analysis of IM-MS-MS data for bread wheat cultivars Chinese Spring (CS) and Hereward using different bread wheat gluten protein sequence databases.

**Protein group**	**No of CS genes/proteins**	**UniProt reviewed prolamins**		**GluPro v 1**		**GluPro v 1.1**		**GluPro v 1.2**	
		**CS**	**Hereward**	**CS**	**Hereward**	**CS**	**Hereward**	**CS**	**Hereward**
Avenin-like	19	0	0	0	0	0	1	0	4
α-gliadins	26	3	6	1	6	2	8	2	7
δ-gliadins	2	0	0	0	0	0	0	1	1
γ-gliadins	11	6	5	6 (5)	9	6	8	7 (5)	8
ω-gliadins	5	0	0	3	8	2	8	2	8
LMW-GS	10	2	3	8 (6)	13	9 (6)	12 (9)	6	11 (7)
HMW-GS	4	5	5	2 (1)	4 (3)	0	4	1 (0)	4
Total	77	16	19	20 (16)	40 (39)	19 (16)	41 (38)	19 (17)	43 (39)

*The number of full length gluten protein sequences in Chinese Spring was recovered from the annotated genome sequence (64) supplemented with the total number of avenin-like sequences from the CS reference proteome available on UniProt (accessed16.01.2020). Databases used in searching were as follows; GluPro v 1 (n = 630), GluPro v 1.1 (n = 685) and GluPro v 1.2 (n = 699). Identifications were made using unique peptides of any length; those with unique peptides ≥5 amino acids in length are given in parentheses*.

Comparison of the number of identifications made with the number of genes present using only genes encoding full length proteins showed that the number of identifications made varied between protein group, being only 7.69% of total α-gliadins compared with 90% of LMW glutenin subunits whilst none of the HMW subunits of glutenin were identified (Table 3). The number of δ-, γ-, ω-gliadins and LMW glutenin subunits matched to gene sequence data was in line with the identifications made by Altenbach et al. (64). The low number of α-gliadins and HMW glutenin subunits identified in Chinese Spring is most likely due to incomplete extraction of the prolamin protein fraction due to lack of aqueous alcohol in the extraction buffer. However, there are some anomalies in the reference proteome since currently it includes sequences for 1Dx5 and 1Dy10 rather than the actual HMW subunits.

There were a number of anomalies regarding the identifications particularly with regards annotation of the HMW glutenin subunits (Table 4, Table S6) and Figure S6). The cvs Chinese Spring and Hereward have well described HMW subunit compositions of 6+8, 2+12, and 7+9, 3+12, respectively. Using the UniProt download five HMW subunits were identified in each cv including an Ax subunit (P02861), despite both cultivars being Glu 1A Null. When the same MS libraries were analysed using the curated sequence databases many of these peptides were no longer identified as being “unique” to one accession, altering the pattern of identifications. For example one unique 15 residue peptide (YPTSPQQSGQGQQGY), which was reproducibly identified with a score of between 5.041 and 5.231 probably arises from the 1Bx subunits in both cvs, as it appears as a tandem repeat in 1Bx sequences including G4Y3Y2 (1Bx7.3), Q6UKZ5 (1Bx14) sequences which share 95.7% sequence identity. Since neither of these sequences are in the reviewed UniProt database, the peptide was mis-identified as being unique to the Ax subunit (P02861). Similar reasons may explain other misidentifications, such as subunits 1Dx5 (P10388) and 1Dy10 (P10387). For example, the unique peptide, QQPGQGQQGHY, was found in the Chinese Spring data set with a score of 6.4 may have originated from a 1By sequence, such as Q52JL2, and was miss assigned to the 1Dy10 subunit again due to the restricted nature of the reviewed UniProt download.

**Table 4:** High molecular weight glutenin subunits identified by IM-MS-MS analysis of bread wheat cvs Chinese Spring (CS) and Hereward.

**Database**	**Cultivar**	**Accession number**	**Subunit type**	**No of peptides (unique peptides)**	**% Sequence coverage**	**Protein score**
UniProt reviewed prolamins	CS	P02861	Ax	2 (1)	26.07	11.08
		P08489	Dx2	44 (7)	50.82	424.10
		P10388	Dx5	40 (1)	29.70	304.93
		P10387	Dy10	18 (3)	24.25	150.34
		P08488	Dy12	20 (5)	38.46	200.36
	Hereward	P02861	Ax	2 (1)	52.48	5.42
		P08489	Dx2	82 (14)	78.76	499.03
		P10388	Dx5	82 (11)	53.24	442.85
		P10387	Dy10	48 (11)	53.09	304.67
		P08488	Dy12	44 (8)	58.01	290.11
GluPro v 1	CS	Q41553	Ax2	11 (1) [0]	19.08	69.83
		G4Y3Y2	Bx7.3	19 (1)	39.49	139.83
	Hereward	A0MZ38	Ax	10 (1) [0]	22.21	65.73
		Q6UKZ5	Bx14	12 (3)	35.58	80.36
		Q52JL2	By	31 (2) [1]	45.63	197.64
		G3FLC7	Dx2/3	48 (1)	57.36	315.01
GluPro v 1.1	CS	None identified	None identified	None identified	None identified	None identified
	Hereward	Q6UKZ5	Bx14	13 (3)	31.90	87.28
		Q52JL2	By	31 (3) [2]	47.83	196.86
		G3FLC7	Dx2/3	47 (1)	56.16	303.04
		Q52JL3	Dy12	22 (1)	46.81	175.38
GluPro v 1.2	CS	A0MZ38	Ax	6 (1) [0]	10.43	50.83
	Hereward	Q6UKZ5	Bx14	15 (5) [3]	46.18	97.57
		Q52JL2	By	28 (2)	46.72	182.53
		G3FLC7	Dx2/3	46 (1)	55.99	293.40
		Q52JL3	Dy12	21 (1)	46.18	170.81

*Identifications of HMW glutenin subunits arising from interrogation of mass spectrometry against different curated databases, the UniProt accession number, subunit type, number of peptides identified, sequence coverage and protein score for that identification. Identifications were made using unique peptides of any length; those with unique peptides ≤ 5 amino acids in length are given in square backets*.

A second factor that affected the sequence accessions identified was that the peptide scores changed with each database. This meant that peptides with scores close to the cut-off of 5.0 were falling in and out of significance. Such a phenomenon probably results from the way in which the decoy database is developed that underpins the reduction of false positive identifications which requires that predicted peptides in the decoy sequence lists are absent from the target sequence list (86). Short motifs, such as those found in the repetitive domain of prolamins, could give rise to ambiguous identifications by appearing in both the decoy and target databases. To take account of this the mass spectra for the unique peptides were visually inspected and included some very short peptides ≤ 5 residues in length, which could map to different proteins. Excluding these peptides reduced the total numbers of gluten proteins identified but did not generally change the nature of the identifications made (Tables 3, 4).

## Discussion

Creation of an expanded gluten protein sequence database has highlighted the large number of partial or fragment sequences and the high degree of redundancy present in UniProt and the NCBI Protein database as well as genome sequences. We also found, as others have observed, that these databases contain sequences that are not always fully annotated, curated or complete, limiting their usefulness for searching MS data (87) including gluten protein proteomics (34). BLAST searching to recover homologous sequences proved important and necessary as this recovered more sequences, especially for *T. turgidum* ssp durum where an additional 45 sequences were identified. Mining of genomes also proved useful for identifying sequences from cereal species, such as *T. turgidum* ssp durum. However, no new sequences were added through mining the rye genome although it only covers the low copy portion representing 2.8Gbp of the total 7.9Gbp, as highly repetitive sequences are difficult to assemble (58). Development of the manually curated databases presented here has addressed these issues and allowed an increased the number of identifications to be made when mining MS data, compared to searching against prolamins in reviewed UniProt.

The number of sequences in the respective cereal species databases correlates well to the number of sequences suggested by genomic and proteomic data (88, 89). Therefore, although the numbers of sequences for barley, rye and oats are relatively low, they should represent almost all of the prolamin sequences that would be observed experimentally. In comparison to ProPepper, GluPro v 6.1 database contains a larger number of sequences attributed to wheat, barley and rye, but fewer for oats. The AllergenOnline database contains fewer sequences from all species because it only includes allergen sequences, which are either IgE-reactive or carry coeliac toxic motifs. Although ω-gliadins have been identified in durum wheat using bread wheat prolamin sequences (71) no ω-gliadins sequences have been attributed to durum wheat in UniProt at present.

Evaluation of coeliac toxicity of prolamins in the GluPro database family using sequences representing T-cell epitopes present in the AllergenOnline database demonstrated that the C hordeins of barley and ω-secalins of rye (both homologues of wheat ω-gliadin) contained, on average, the highest number, density and percentage coverage by coeliac toxic motifs. Interestingly, the 75 k γ-secalins, like the γ-gliadins in both bread wheat and *T. turgidum* ssp durum (50, 71), also contained a relatively high number of coeliac toxic motifs, in contrast to both the 40 k γ-secalins and γ-hordeins. As the 75 k γ-secalins comprise ~50% of the total seed proteins of rye, this could pose a high risk to individuals with coeliac disease (52). Unlike barley and rye, there was little variation in the potential coeliac toxicity of the gliadins between bread wheat and *T. turgidum* ssp durum (50). Gliadins from bread wheat are often considered the most coeliac toxic group, with a 33 mer peptide derived from α2-gliadin described at the most important coeliac toxic fragment (90, 91). In addition to the content of coeliac toxic motifs, the total prolamin content and proportions of each prolamin type within a given cereal species needs to be taken into account in assessing potential coeliac toxicity. For example, although ω-type prolamins (ω-gliadins, ω-secalins and C hordeins) contained a relatively large number of coeliac toxic motifs, these proteins only constitute a minor fraction of total expressed prolamins in these grains. In contrast, the LMW glutenin fraction present in bread wheat, *T. turgidum* ssp durum and barley could pose a greater risk to coeliac sufferers, as although they contain fewer coeliac toxic motifs, they account for ~30% of total seed storage prolamins (92).

The avenin-like proteins from bread wheat *T. turgidum* ssp durum and barley did not contain any known coeliac toxic motifs, and consequently may pose little or no risk to those with coeliac disease. However, this will require confirmation through, for example, assessing the capacity of these proteins to stimulate T-cells. In contrast, avenin proteins from oats contained many coeliac toxic motifs. In addition, since avenins comprise the minor fraction of seed storage proteins in oats further reducing the total content of coeliac toxic motifs in oats compared to wheat, barley and rye. This supports observations that oats cannot be tolerated by some of those individuals with coeliac disease (19) and calls in to question claim that oats should be included in a gluten-free diet. With regards IgE-mediated food allergy, only seven of the eleven sequence accessions corresponding to seed storage prolamin food allergens mapped to full length protein sequences in the GluPro databases. Several prolamins contained IgE epitopes identified by Juhasz et al. (93), particularly in the ω-type prolamins from all the cereal species except oats, with one epitope (QQFPQQQ) only being present in bread wheat and *T. turgidum* ssp durum.

The development of a suite of curated prolamin sequences from bread wheat, *T. turgidum* ssp durum, barley, rye and oats into a family of databases will support mining of mass spectrometric data in future. It will also potentially provide the protein level evidence currently lacking for protein sequences contained in the databases, such as the α-prolamins in rye, avenin-like proteins, and δ-gliadin in *T. turgidum* ssp durum, avenin-like b proteins in barley and A-avenins in oats. The mapping of coeliac toxic motifs within the database will allow peptide markers for coeliac toxic motifs to be identified using mass spectrometry. This could thereby support the development of new analytical methods, which can quantify the burden of toxic motifs in gluten-containing and gluten-free food.

## Data Availability Statement

The curated sequence sets are available for download in FASTA format through the Figshare data repository (doi: 10.6084/m9.figshare.12613154).

## Author Contributions

MD generated the databases and completed the phylogenetic analysis of the sequences along with coeliac toxic motif evaluation. SB undertook proteomic analysis of wheat grain samples. MD and EM conceived and wrote the manuscript. CN, PS, and LG contributed to interpretation and discussion of data generated and EM wrote the manuscript and revision of the paper.

## Conflict of Interest

LG is employed by Waters Corporation, a manufacturer and vendor of mass spectrometers used for proteomics. The remaining authors declare that the research was conducted in the absence of any commercial or financial relationships that could be construed as a potential conflict of interest.
