# Supplementary material for: Mapping Coeliac Toxic Motifs in the Prolamin Seed Storage Proteins of Barley, Rye, and Oats Using a Curated Sequence Database
Source: Front Nutr. 2020 Jul 17;7:87. doi: 10.3389/fnut.2020.00087 (PMC7379453; doi:10.3389/fnut.2020.00087)
Supplement: Supplementary file 1 [file Table_1.DOCX]

**Table S1. UniProt accession number of all *T. turgidum* ssp durum sequences contained in the GluPro v 2.0 database.** The prolamin classification arising from sequence analysis is provided together with within-group sequence homology. Supporting literature is cited below the table.

| **UniProt accession number or identifier** | **Evidence level** | **Supporting literature reference** | **GluPro classification** | **Sequence homology** |
| --- | --- | --- | --- | --- |
| 182939 | Genome | ([Maccaferri et al., 2019](#_ENREF_10)) | α-gliadin | 90.24% |
| 183774 | Genome | ([Maccaferri et al., 2019](#_ENREF_10)) |  |  |
| 183775 | Genome | ([Maccaferri et al., 2019](#_ENREF_10)) |  |  |
| 183776 | Genome | ([Maccaferri et al., 2019](#_ENREF_10)) |  |  |
| 183778 | Genome | ([Maccaferri et al., 2019](#_ENREF_10)) |  |  |
| 183924 | Genome | ([Maccaferri et al., 2019](#_ENREF_10)) |  |  |
| 183925 | Genome | ([Maccaferri et al., 2019](#_ENREF_10)) |  |  |
| 183926 | Genome | ([Maccaferri et al., 2019](#_ENREF_10)) |  |  |
| 187277 | Genome | ([Maccaferri et al., 2019](#_ENREF_10)) |  |  |
| 187279 | Genome | ([Maccaferri et al., 2019](#_ENREF_10)) |  |  |
| A0A0E3UQW2 | Genome | ([Ozuna et al., 2015](#_ENREF_12)) |  |  |
| A0A0E3UQW4 | Genome | ([Ozuna et al., 2015](#_ENREF_12)) |  |  |
| A0A0E3UQW5 | Genome | ([Ozuna et al., 2015](#_ENREF_12)) |  |  |
| A0A0E3UR92 | Genome | ([Ozuna et al., 2015](#_ENREF_12)) |  |  |
| A0A0E3UR95 | Genome | ([Ozuna et al., 2015](#_ENREF_12)) |  |  |
| A0A0E3UR97 | Genome | ([Ozuna et al., 2015](#_ENREF_12)) |  |  |
| A0A0E3UR98 | Genome | ([Ozuna et al., 2015](#_ENREF_12)) |  |  |
| A0A0E3Z552 | Protein | ([Pilolli et al., 2019](#_ENREF_13)) |  |  |
| A0A0E3Z559 | Genome | ([Ozuna et al., 2015](#_ENREF_12)) |  |  |
| A0A0E3Z563 | Genome | ([Ozuna et al., 2015](#_ENREF_12)) |  |  |
| A0A0E3Z5B3 | Genome | ([Ozuna et al., 2015](#_ENREF_12)) |  |  |
| A0A0E3Z5B6 | Genome | ([Ozuna et al., 2015](#_ENREF_12)) |  |  |
| A0A0E3Z5Z1 | Genome | ([Ozuna et al., 2015](#_ENREF_12)) |  |  |
| A0A0E3Z5Z6 | Genome | ([Ozuna et al., 2015](#_ENREF_12)) |  |  |
| A0A0E3Z6Q1 | Genome | ([Ozuna et al., 2015](#_ENREF_12)) |  |  |
| A0A0E3Z6Q7 | Genome | ([Ozuna et al., 2015](#_ENREF_12)) |  |  |
| A0A0E3Z7J9 | Genome | ([Ozuna et al., 2015](#_ENREF_12)) |  |  |
| A0A0E3Z7K2 | Protein | ([Pilolli et al., 2019](#_ENREF_13)) |  |  |
| A0A446V2H3 | Genome | Manual submission L. Milanese Sep 2017 |  |  |
| A0A446V2H9 | Genome | Manual submission L. Milanese Sep 2017 |  |  |
| A0A446V2I3 | Genome | Manual submission L. Milanese Sep 2017 |  |  |
| A0A446V2I9 | Genome | Manual submission L. Milanese Sep 2017 |  |  |
| A0A446V2J2 | Genome | Manual submission L. Milanese Sep 2017 |  |  |
| A0A446V2J5 | Genome | Manual submission L. Milanese Sep 2017 |  |  |
| A0A446V2J7 | Genome | Manual submission L. Milanese Sep 2017 |  |  |
| A0A446V2J8 | Genome | Manual submission L. Milanese Sep 2017 |  |  |
| A0A446V2J9 | Genome | Manual submission L. Milanese Sep 2017 |  |  |
| A0A446V2K2 | Genome | Manual submission L. Milanese Sep 2017 |  |  |
| A0A446V2K5 | Genome | Manual submission L. Milanese Sep 2017 |  |  |
| A0A446V2K7 | Genome | Manual submission L. Milanese Sep 2017 |  |  |
| A0A446V2K9 | Genome | Manual submission L. Milanese Sep 2017 |  |  |
| A0A446V2L4 | Genome | Manual submission L. Milanese Sep 2017 |  |  |
| A0A446V2L5 | Genome | Manual submission L. Milanese Sep 2017 |  |  |
| A0A446V2L7 | Genome | Manual submission L. Milanese Sep 2017 |  |  |
| A0A446V2L8 | Genome | Manual submission L. Milanese Sep 2017 |  |  |
| A0A446V2M4 | Genome | Manual submission L. Milanese Sep 2017 |  |  |
| A0A446V2M7 | Genome | Manual submission L. Milanese Sep 2017 |  |  |
| A0A446V2M9 | Genome | Manual submission L. Milanese Sep 2017 |  |  |
| A0A446V2N5 | Genome | Manual submission L. Milanese Sep 2017 |  |  |
| A0A446V2P0 | Genome | Manual submission L. Milanese Sep 2017 |  |  |
| A0A446V2P5 | Genome | Manual submission L. Milanese Sep 2017 |  |  |
| A0A446V2Q0 | Genome | Manual submission L. Milanese Sep 2017 |  |  |
| A0A446V2Q9 | Genome | Manual submission L. Milanese Sep 2017 |  |  |
| A0A446W085 | Genome | Manual submission L. Milanese Sep 2017 |  |  |
| A0A446W087 | Genome | Manual submission L. Milanese Sep 2017 |  |  |
| A0A446W089 | Genome | Manual submission L. Milanese Sep 2017 |  |  |
| A0A446W093 | Genome | Manual submission L. Milanese Sep 2017 |  |  |
| A0A446W094 | Genome | Manual submission L. Milanese Sep 2017 |  |  |
| A0A446W095 | Genome | Manual submission L. Milanese Sep 2017 |  |  |
| A0A446W096 | Genome | Manual submission L. Milanese Sep 2017 |  |  |
| A0A446W099 | Genome | Manual submission L. Milanese Sep 2017 |  |  |
| A0A446W0A1 | Genome | Manual submission L. Milanese Sep 2017 |  |  |
| A0A446W0A3 | Genome | Manual submission L. Milanese Sep 2017 |  |  |
| A0A446W0A5 | Genome | Manual submission L. Milanese Sep 2017 |  |  |
| A0A446W0A6 | Genome | Manual submission L. Milanese Sep 2017 |  |  |
| A0A446W0A9 | Genome | Manual submission L. Milanese Sep 2017 |  |  |
| A0A446W0B2 | Genome | Manual submission L. Milanese Sep 2017 |  |  |
| A0A446W0B4 | Genome | Manual submission L. Milanese Sep 2017 |  |  |
| A0A446W0B5 | Genome | Manual submission L. Milanese Sep 2017 |  |  |
| A0A446W0B7 | Genome | Manual submission L. Milanese Sep 2017 |  |  |
| A0A446W0B9 | Genome | Manual submission L. Milanese Sep 2017 |  |  |
| A0A446W0C2 | Genome | Manual submission L. Milanese Sep 2017 |  |  |
| A0A446W0C7 | Genome | Manual submission L. Milanese Sep 2017 |  |  |
| A0A446W0C8 | Genome | Manual submission L. Milanese Sep 2017 |  |  |
| A0A446W0C9 | Genome | Manual submission L. Milanese Sep 2017 |  |  |
| A0A446W0D4 | Genome | Manual submission L. Milanese Sep 2017 |  |  |
| A0A446W0D7 | Genome | Manual submission L. Milanese Sep 2017 |  |  |
| A0A446W0D8 | Genome | Manual submission L. Milanese Sep 2017 |  |  |
| A0A446W0E7 | Genome | Manual submission L. Milanese Sep 2017 |  |  |
| A0A446W0E8 | Genome | Manual submission L. Milanese Sep 2017 |  |  |
| A0A446W0F5 | Genome | Manual submission L. Milanese Sep 2017 |  |  |
| A0A446W0F7 | Genome | Manual submission L. Milanese Sep 2017 |  |  |
| A0A446W0G6 | Genome | Manual submission L. Milanese Sep 2017 |  |  |
| A0A446W0H6 | Genome | Manual submission L. Milanese Sep 2017 |  |  |
| A0A446W0I4 | Genome | Manual submission L. Milanese Sep 2017 |  |  |
| A0A446W0J7 | Genome | Manual submission L. Milanese Sep 2017 |  |  |
| A0A446W0K7 | Genome | Manual submission L. Milanese Sep 2017 |  |  |
| A0A446W0L8 | Genome | Manual submission L. Milanese Sep 2017 |  |  |
| A0A446W0N0 | Genome | Manual submission L. Milanese Sep 2017 |  |  |
| A0A446W1C0 | Genome | Manual submission L. Milanese Sep 2017 |  |  |
| A0A446W1C2 | Genome | Manual submission L. Milanese Sep 2017 |  |  |
| A0A446W1C4 | Genome | Manual submission L. Milanese Sep 2017 |  |  |
| A0A446W1D6 | Genome | Manual submission L. Milanese Sep 2017 |  |  |
| A0A446W1F2 | Genome | Manual submission L. Milanese Sep 2017 |  |  |
| A0A446W1F6 | Genome | Manual submission L. Milanese Sep 2017 |  |  |
| A0A446W1H7 | Genome | Manual submission L. Milanese Sep 2017 |  |  |
| A0A446W1J4 | Genome | Manual submission L. Milanese Sep 2017 |  |  |
| D2X6C8 | Genomic DNA | ([Gregorini et al., 2009](#_ENREF_7)) |  |  |
| D2X6C9 | Protein | ([Pilolli et al., 2019](#_ENREF_13)) |  |  |
| D2X6D0 | Genomic DNA | ([Gregorini et al., 2009](#_ENREF_7)) |  |  |
| D2X6D1 | Genomic DNA | ([Gregorini et al., 2009](#_ENREF_7)) |  |  |
| D2X6D2 | Genomic DNA | ([Gregorini et al., 2009](#_ENREF_7)) |  |  |
| D2X6D3 | Genomic DNA | ([Gregorini et al., 2009](#_ENREF_7)) |  |  |
| D2X6D4 | Genomic DNA | ([Gregorini et al., 2009](#_ENREF_7)) |  |  |
| D2X6D5 | Genomic DNA | ([Gregorini et al., 2009](#_ENREF_7)) |  |  |
| D2X6D8 | Genomic DNA | ([Gregorini et al., 2009](#_ENREF_7)) |  |  |
| D2X6D9 | Genomic DNA | ([Gregorini et al., 2009](#_ENREF_7)) |  |  |
| Q2V5Z6 | Genomic DNA | ([H.-Y. Wang et al., 2007](#_ENREF_17)) |  |  |
| Q2V5Z7 | Genomic DNA | 10.1016/S1671-2927(07)60013-3 ([H.-Y. Wang et al., 2007](#_ENREF_17)) |  |  |
| Q5NDA5 | mRNA | Manual submission Cifarelli R.A. Dec 2004 |  |  |
| 182970 | Genome | ([Maccaferri et al., 2019](#_ENREF_10)) | Avenin-like | 67.89% |
| 185636 | Genome | ([Maccaferri et al., 2019](#_ENREF_10)) |  |  |
| 186240 | Genome | ([Maccaferri et al., 2019](#_ENREF_10)) |  |  |
| 186241 | Genome | ([Maccaferri et al., 2019](#_ENREF_10)) |  |  |
| 186242 | Genome | ([Maccaferri et al., 2019](#_ENREF_10)) |  |  |
| A0A446RKG6 | Genome | Manual submission L. Milanese Sep 2017 |  |  |
| A0A446RKK3 | Genome | Manual submission L. Milanese Sep 2017 |  |  |
| A0A446RL10 | Genome | Manual submission L. Milanese Sep 2017 |  |  |
| A0A446WXR1 | Genome | Manual submission L. Milanese Sep 2017 |  |  |
| A0A446WXR7 | Genome | Manual submission L. Milanese Sep 2017 |  |  |
| A0A446WXS8 | Genome | Manual submission L. Milanese Sep 2017 |  |  |
| A0A446WXT0 | Genome | Manual submission L. Milanese Sep 2017 |  |  |
| A0A446WXT3 | Genome | Manual submission L. Milanese Sep 2017 |  |  |
| A0A446WXT5 | Genome | Manual submission L. Milanese Sep 2017 |  |  |
| A0A446WXV0 | Genome | Manual submission L. Milanese Sep 2017 |  |  |
| A0A446WXW6 | Genome | Manual submission L. Milanese Sep 2017 |  |  |
| 184653 | Genome | ([Maccaferri et al., 2019](#_ENREF_10)) |  |  |
| 184654 | Genome | ([Maccaferri et al., 2019](#_ENREF_10)) |  |  |
| 184655 | Genome | ([Maccaferri et al., 2019](#_ENREF_10)) |  |  |
| 184656 | Genome | ([Maccaferri et al., 2019](#_ENREF_10)) |  |  |
| 188204 | Genome | ([Maccaferri et al., 2019](#_ENREF_10)) |  |  |
| A0A446WXS7 | Genome | Manual submission L. Milanese Sep 2017 |  |  |
| A0A446IHB0 | Genome | Manual submission L. Milanese Sep 2017 | δ-gliadin | 99.29% |
| A0A446IHE0 | Genome | Manual submission L. Milanese Sep 2017 |  |  |
| A0A446IHB5 | Genome | Manual submission L. Milanese Sep 2017 | γ-gliadin | 86.73% |
| A0A446IHB8 | Genome | Manual submission L. Milanese Sep 2017 |  |  |
| A0A446IHC0 | Genome | Manual submission L. Milanese Sep 2017 |  |  |
| A0A446IHC1 | Genome | Manual submission L. Milanese Sep 2017 |  |  |
| A0A446IHC3 | Genome | Manual submission L. Milanese Sep 2017 |  |  |
| A0A446IHC5 | Genome | Manual submission L. Milanese Sep 2017 |  |  |
| A0A446IHC7 | Genome | Manual submission L. Milanese Sep 2017 |  |  |
| A0A446IHD2 | Genome | Manual submission L. Milanese Sep 2017 |  |  |
| A0A446IHD5 | Genome | Manual submission L. Milanese Sep 2017 |  |  |
| A0A446IHD6 | Genome | Manual submission L. Milanese Sep 2017 |  |  |
| A0A446IHD7 | Genome | Manual submission L. Milanese Sep 2017 |  |  |
| A0A446IHE6 | Genome | Manual submission L. Milanese Sep 2017 |  |  |
| A0A446IHF4 | Genome | Manual submission L. Milanese Sep 2017 |  |  |
| A0A446IHJ1 | Genome | Manual submission L. Milanese Sep 2017 |  |  |
| A0A446IHK0 | Genome | Manual submission L. Milanese Sep 2017 |  |  |
| A0A446JG43 | Genome | Manual submission L. Milanese Sep 2017 |  |  |
| A0A446JG55 | Genome | Manual submission L. Milanese Sep 2017 |  |  |
| A0A446JG61 | Genome | Manual submission L. Milanese Sep 2017 |  |  |
| A0A446JG74 | Genome | Manual submission L. Milanese Sep 2017 |  |  |
| A0A446JG83 | Genome | Manual submission L. Milanese Sep 2017 |  |  |
| A0A446JG84 | Genome | Manual submission L. Milanese Sep 2017 |  |  |
| A0A446JG85 | Genome | Manual submission L. Milanese Sep 2017 |  |  |
| A0A446JG88 | Genome | Manual submission L. Milanese Sep 2017 |  |  |
| A0A446JG98 | Genome | Manual submission L. Milanese Sep 2017 |  |  |
| A0A446JG99 | Genome | Manual submission L. Milanese Sep 2017 |  |  |
| A0A446JGA9 | Genome | Manual submission L. Milanese Sep 2017 |  |  |
| A0A446JGE5 | Genome | Manual submission L. Milanese Sep 2017 |  |  |
| A0A446JGF8 | Genome | Manual submission L. Milanese Sep 2017 |  |  |
| A0A446JGQ5 | Genome | Manual submission L. Milanese Sep 2017 |  |  |
| A0A446JGQ8 | Genome | Manual submission L. Milanese Sep 2017 |  |  |
| Q41602 | Genomic DNA | ([R D'Ovidio, Tanzarella, & Porceddu, 1991](#_ENREF_1)) |  |  |
| Q6EEW3 | cDNA | ([Pistón et al., 2006](#_ENREF_14)) |  |  |
| Q6EEW4 | cDNA | ([Pistón et al., 2006](#_ENREF_14)) |  |  |
| Q6EEW5 | Protein | ([Pilolli et al., 2019](#_ENREF_13)) |  |  |
| Q6EEW6 | cDNA | ([Pistón et al., 2006](#_ENREF_14)) |  |  |
| Q84M19 | Protein | ([Visioli et al., 2016](#_ENREF_16)) |  |  |
| A0A0E4G9A4 | Protein | ([Santagati et al., 2016](#_ENREF_15)) | HMW glutenin subunit | 85.46% |
| A0A2L1K3K6 | Protein | ([Pilolli et al., 2019](#_ENREF_13)) |  |  |
| K4N1X7 | Genomic DNA | ([Jiang et al., 2012](#_ENREF_8)) |  |  |
| Q6UJY5 | Genomic DNA | ([Kong et al., 2004](#_ENREF_9)) |  |  |
| Q6UJY7 | Genomic DNA | ([Kong et al., 2004](#_ENREF_9)) |  |  |
| Q84TG6 | Genomic DNA | Direct submission Jiang Y., Sun M., Zheng J., Xiao Y., Yan Y. March 2003 |  |  |
| Q8RVX0 | Protein | ([Pilolli et al., 2019](#_ENREF_13)) |  |  |
| *CAA36063.1 | mRNA | Manual submission B. G Cassidy, J. Dvorak March 1995 | LMW glutenin subunit | 81.86% |
| 185860 | Genome | ([Maccaferri et al., 2019](#_ENREF_10)) |  |  |
| 185861 | Genome | ([Maccaferri et al., 2019](#_ENREF_10)) |  |  |
| 185862 | Genome | ([Maccaferri et al., 2019](#_ENREF_10)) |  |  |
| 185863 | Genome | ([Maccaferri et al., 2019](#_ENREF_10)) |  |  |
| 185864 | Genome | ([Maccaferri et al., 2019](#_ENREF_10)) |  |  |
| 185866 | Genome | ([Maccaferri et al., 2019](#_ENREF_10)) |  |  |
| 185867 | Genome | ([Maccaferri et al., 2019](#_ENREF_10)) |  |  |
| 185868 | Genome | ([Maccaferri et al., 2019](#_ENREF_10)) |  |  |
| A0A2P1BXV0 | Protein | ([Pilolli et al., 2019](#_ENREF_13)) |  |  |
| A0A446IHD8 | Genome | Manual submission L. Milanese Sep 2017 |  |  |
| A0A446IHD9 | Genome | Manual submission L. Milanese Sep 2017 |  |  |
| A0A446IHE3 | Genome | Manual submission L. Milanese Sep 2017 |  |  |
| A0A446IHF1 | Genome | Manual submission L. Milanese Sep 2017 |  |  |
| A0A446IHF7 | Genome | Manual submission L. Milanese Sep 2017 |  |  |
| A0A446IHH8 | Genome | Manual submission L. Milanese Sep 2017 |  |  |
| A0A446IHK2 | Genome | Manual submission L. Milanese Sep 2017 |  |  |
| A0A446IHK8 | Genome | Manual submission L. Milanese Sep 2017 |  |  |
| A0A446IHQ7 | Genome | Manual submission L. Milanese Sep 2017 |  |  |
| A7XDG0 | Protein | ([Pilolli et al., 2019](#_ENREF_13)) |  |  |
| D5FPE1 | Protein | ([Visioli et al., 2016](#_ENREF_16)) |  |  |
| D5FPE4 | Genomic DNA | ([L. H. Wang et al., 2010](#_ENREF_18)) |  |  |
| G9AYK6 | Genomic DNA | Manual submission Sestili F., Masci S., Lafiandra D. Dec 2011 |  |  |
| O49958 | Genomic DNA | ([D’Ovidio et al., 1997](#_ENREF_4)) |  |  |
| Q0Q2J0 | Genomic DNA | ([Gao et al., 2007](#_ENREF_6)) |  |  |
| Q0Q2J1 | Genomic DNA | ([Gao et al., 2007](#_ENREF_6)) |  |  |
| Q41603 | Genomic DNA | ([R. D'Ovidio, Tanzarella, & Porceddu, 1992](#_ENREF_2)) |  |  |
| Q68VI0 | Genomic DNA | Manual submission Oak M.D. Jan 2003 |  |  |
| Q84NE4 | Genomic DNA | ([Wicker et al., 2003](#_ENREF_19)) |  |  |
| Q9FEQ1 | Protein | ([Mamone et al., 2009](#_ENREF_11)) |  |  |
| Q9FEQ2 | Protein | ([Ferreira et al., 2014](#_ENREF_5)) |  |  |
| Q9XGE9 | Genomic DNA | ([D’Ovidio et al., 1999](#_ENREF_3)) |  |  |
| Q9XGF0 | Genomic DNA | ([D’Ovidio et al., 1999](#_ENREF_3)) |  |  |

**References**

D'Ovidio, R., Tanzarella, O. A., & Porceddu, E. (1991). Cloning and sequencing of a PCR amplified gamma-gliadin gene from durum wheat (Triticum turgidum (L.) Thell. conv. durum (Desf.) MK.). *Plant Science, 75*(2), 229-236. doi: 10.1016/0168-9452(91)90238-4

D'Ovidio, R., Tanzarella, O. A., & Porceddu, E. (1992). Nucleotide sequence of a low-molecular-weight glutenin from Triticum durum. *Plant Mol Biol, 18*(4), 781-784. doi: 10.1007/bf00020020

D’Ovidio, R., Marchitelli, C., Ercoli Cardelli, L., & Porceddu, E. (1999). Sequence similarity between allelic Glu-B3 genes related to quality properties of durum wheat. *Theoretical and Applied Genetics, 98*(3-4), 455-461. doi: 10.1007/s001220051091

D’Ovidio, R., Simeone, M., Masci, S., & Porceddu, E. (1997). Molecular characterization of a LMW-GS gene located on chromosome 1B and the development of primers specific for the Glu-B3 complex locus in durum wheat. *Theoretical and Applied Genetics, 95*(7), 1119-1126. doi: 10.1007/s001220050671

Ferreira, M. S. L., Mangavel, C., Rogniaux, H., Bonicel, J., Samson, M.-F., & Morel, M.-H. (2014). A MALDI-TOF based study of the in-vivo assembly of glutenin polymers of durum wheat. *Food Research International, 63*, 89-99. doi: 10.1016/j.foodres.2013.12.025

Gao, S., Gu, Y. Q., Wu, J., Coleman-Derr, D., Huo, N., Crossman, C., Jia, J., Zuo, Q., Ren, Z., Anderson, O. D., & Kong, X. (2007). Rapid evolution and complex structural organization in genomic regions harboring multiple prolamin genes in the polyploid wheat genome. *Plant Mol Biol, 65*(1-2), 189-203. doi: 10.1007/s11103-007-9208-1

Gregorini, A., Colomba, M., Ellis, H. J., & Ciclitira, P. J. (2009). Immunogenicity characterization of two ancient wheat alpha-gliadin peptides related to coeliac disease. *Nutrients, 1*(2), 276-290. doi: 10.3390/nu1020276

Jiang, Q. T., Ma, J., Zhao, S., Zhao, Q. Z., Lan, X. J., Dai, S. F., Lu, Z. X., Zheng, Y. L., & Wei, Y. M. (2012). Characterization of HMW-GSs and their gene inaction in tetraploid wheat. *Genetica, 140*(7-9), 325-335. doi: 10.1007/s10709-012-9683-4

Kong, X. Y., Gu, Y. Q., You, F. M., Dubcovsky, J., & Anderson, O. D. (2004). Dynamics of the evolution of orthologous and paralogous portions of a complex locus region in two genomes of allopolyploid wheat. *Plant Mol Biol, 54*(1), 55-69. doi: 10.1023/B:PLAN.0000028768.21587.dc

Maccaferri, M., Harris, N. S., Twardziok, S. O., Pasam, R. K., Gundlach, H., Spannagl, M., Ormanbekova, D., Lux, T., Prade, V. M., Milner, S. G., Himmelbach, A., Mascher, M., Bagnaresi, P., Faccioli, P., Cozzi, P., Lauria, M., Lazzari, B., Stella, A., Manconi, A., Gnocchi, M., Moscatelli, M., Avni, R., Deek, J., Biyiklioglu, S., Frascaroli, E., Corneti, S., Salvi, S., Sonnante, G., Desiderio, F., Mare, C., Crosatti, C., Mica, E., Ozkan, H., Kilian, B., De Vita, P., Marone, D., Joukhadar, R., Mazzucotelli, E., Nigro, D., Gadaleta, A., Chao, S., Faris, J. D., Melo, A. T. O., Pumphrey, M., Pecchioni, N., Milanesi, L., Wiebe, K., Ens, J., MacLachlan, R. P., Clarke, J. M., Sharpe, A. G., Koh, C. S., Liang, K. Y. H., Taylor, G. J., Knox, R., Budak, H., Mastrangelo, A. M., Xu, S. S., Stein, N., Hale, I., Distelfeld, A., Hayden, M. J., Tuberosa, R., Walkowiak, S., Mayer, K. F. X., Ceriotti, A., Pozniak, C. J., & Cattivelli, L. (2019). Durum wheat genome highlights past domestication signatures and future improvement targets. *Nat Genet, 51*(5), 885-895. doi: 10.1038/s41588-019-0381-3

Mamone, G., De Caro, S., Di Luccia, A., Addeo, F., & Ferranti, P. (2009). Proteomic-based analytical approach for the characterization of glutenin subunits in durum wheat. *J Mass Spectrom, 44*(12), 1709-1723. doi: 10.1002/jms.1680

Ozuna, C. V., Iehisa, J. C., Gimenez, M. J., Alvarez, J. B., Sousa, C., & Barro, F. (2015). Diversification of the celiac disease alpha-gliadin complex in wheat: a 33-mer peptide with six overlapping epitopes, evolved following polyploidization. *Plant J, 82*(5), 794-805. doi: 10.1111/tpj.12851

Pilolli, R., Gadaleta, A., Di Stasio, L., Lamonaca, A., De Angelis, E., Nigro, D., De Angelis, M., Mamone, G., & Monaci, L. (2019). A Comprehensive Peptidomic Approach to Characterize the Protein Profile of Selected Durum Wheat Genotypes: Implication for Coeliac Disease and Wheat Allergy. *Nutrients, 11*(10). doi: 10.3390/nu11102321

Pistón, F., Dorado, G., Martín, A., & Barro, F. (2006). Cloning of nine γ-gliadin mRNAs (cDNAs) from wheat and the molecular characterization of comparative transcript levels of γ-gliadin subclasses. *Journal of Cereal Science, 43*(1), 120-128. doi: 10.1016/j.jcs.2005.07.002

Santagati, V. D., Sestili, F., Lafiandra, D., D'Ovidio, R., Rogniaux, H., & Masci, S. (2016). Characterization of durum wheat high molecular weight glutenin subunits Bx20 and By20 sequences by a molecular and proteomic approach. *J Mass Spectrom, 51*(7), 512-517. doi: 10.1002/jms.3776

Visioli, G., Galieni, A., Stagnari, F., Bonas, U., Speca, S., Faccini, A., Pisante, M., & Marmiroli, N. (2016). Proteomics of Durum Wheat Grain during Transition to Conservation Agriculture. *PLoS One, 11*(6), e0156007. doi: 10.1371/journal.pone.0156007

Wang, H.-Y., Wei, Y.-M., Ze, H.-Y., & Zheng, Y.-L. (2007). Isolation and Analysis of α-Gliadin Gene Coding Sequences from Triticum durum. *Agricultural Sciences in China, 6*(1), 25-32. doi: 10.1016/s1671-2927(07)60013-3

Wang, L. H., Zhou, M., Li, H. L., He, Z. H., & Xia, X. C. (2010). Cloning and phylogenetic analysis of low-molecular-weight glutenin subunit genes at Glu-B3 locus in common wheat relative species. *Yi Chuan, 32*(6), 613-624. doi: 10.3724/sp.j.1005.2010.00613

Wicker, T., Yahiaoui, N., Guyot, R., Schlagenhauf, E., Liu, Z. D., Dubcovsky, J., & Keller, B. (2003). Rapid genome divergence at orthologous low molecular weight glutenin loci of the A and Am genomes of wheat. *Plant Cell, 15*(5), 1186-1197. doi: 10.1105/tpc.011023
