# Supplementary material for: Mapping Coeliac Toxic Motifs in the Prolamin Seed Storage Proteins of Barley, Rye, and Oats Using a Curated Sequence Database
Source: Front Nutr. 2020 Jul 17;7:87. doi: 10.3389/fnut.2020.00087 (PMC7379453; doi:10.3389/fnut.2020.00087)
Supplement: Supplementary file 2 [file Table_2.DOCX]

| **UniProt accession number** | **Evidence level** | **Supporting literature** | **GluPro Classification** | **Sequence similarity** |
| --- | --- | --- | --- | --- |
| A0A287JK04 | Genome | 10.1038/nature11543 | Avenin-like | 54.27%  **Table S2. UniProt accession number of all *H. vulgare* sequences contained in the GluPro v 3.0 database**. The prolamin classification arising from sequence analysis is provided together with within-group sequence homology. |
| M0V4S8 | Genome | 10.1038/nature11543 |  |  |
| M0VEH1 | Genome | 10.1038/nature11543 |  |  |
| F2EGD5 | Protein | https://doi.org/10.1016/j.foodchem.2014.09.012 |  |  |
| M0VH55 | Genome | 10.1038/nature11543 |  |  |
| A7XUQ7 | Genomic translation | 10.1016/j.jcs.2008.04.002 |  |  |
| M0VWJ3 | Genome | 10.1038/nature11543 |  |  |
| I6TMW0 | Protein | https://doi.org/10.1371/journal.pone.0172819 | B1 hordein | 90.35% |
| I6QM99 | mRNA | 10.1186/1471-2229-12-184 |  |  |
| I6SW25 | Protein | https://doi.org/10.1371/journal.pone.0172819 |  |  |
| Q40021 | Protein | https://doi.org/10.1371/journal.pone.0172819 |  |  |
| I6R4A7 | Protein | https://doi.org/10.1371/journal.pone.0172819 |  |  |
| I6TRT2 | Protein | https://doi.org/10.1371/journal.pone.0172819 |  |  |
| I6SJ22 | Protein | https://doi.org/10.1371/journal.pone.0172819 |  |  |
| Q40026 | Genomic translation | 10.1007/BF00040661 |  |  |
| Q3LTR1 | Genomic translation | 10.1134/S0026893308010081 |  |  |
| I6TMV2 | mRNA | 10.1186/1471-2229-12-184 |  |  |
| P06470 | Protein | 10.1093/nar/13.20.7327 https://doi.org/10.1371/journal.pone.0172819 |  |  |
| A0A287EFE1 | Genome | 10.1038/nature11543 |  |  |
| I6SJ13 | Protein | https://doi.org/10.1371/journal.pone.0172819 |  |  |
| A0A287EFF7 | Genome | 10.1038/nature11543 |  |  |
| A0A287EFH8 | Genome | 10.1038/nature11543 |  |  |
| Q2XQF0 | Genomic translation | 10.1134/S0026893308010081 |  |  |
| A0A287EFD4 | Genome | 10.1038/nature11543 |  |  |
| A0A287EFB3 | Genome | 10.1038/nature11543 |  |  |
| A0A287EFG2 | Genome | 10.1038/nature11543 |  |  |
| A0A0K2GRS6 | Genomic translation |  |  |  |
| Q3YAF9 | Protein | https://doi.org/10.1371/journal.pone.0172819 |  |  |
| A0A287EIW5 | mRNA | 10.1186/1471-2229-12-184 | B3 hordein | 91.33% |
| Q0PIV6 | Protein | https://doi.org/10.1371/journal.pone.0172819 |  |  |
| A0A287EIZ3 | Genome | 10.1038/nature11543 |  |  |
| I6SW30 | Protein | https://doi.org/10.1371/journal.pone.0172819 |  |  |
| I6TMW4 | mRNA | 10.1186/1471-2229-12-184 |  |  |
| I6SJ26 | Protein | https://doi.org/10.1371/journal.pone.0172819 |  |  |
| I6TRT5 | Protein | https://doi.org/10.1371/journal.pone.0172819 |  |  |
| I6TEV5 | Protein | https://doi.org/10.1371/journal.pone.0172819 |  |  |
| A0A287EJ06 | Genome | 10.1038/nature11543 |  |  |
| C7FB15 | Genomic translation | 10.1007/s10709-009-9415-6 |  |  |
| C7FB13 | Protein | https://doi.org/10.1371/journal.pone.0172819 |  |  |
| C7FB14 | Protein | https://doi.org/10.1371/journal.pone.0172819 |  |  |
| Q2XQF1 | Protein | https://doi.org/10.1371/journal.pone.0172819 |  |  |
| C7FB16 | Genomic translation | 10.1007/s10709-009-9415-6 |  |  |
| C7FB17 | Protein | https://doi.org/10.1371/journal.pone.0172819 |  |  |
| A0A287EIM7 | Protein | https://doi.org/10.1371/journal.pone.0172819 | C hordein | 69.76% |
| A0A287EEX5 | Genome | 10.1038/nature11543 |  |  |
| Q41210 | Protein | https://doi.org/10.1371/journal.pone.0172819 |  |  |
| I6TEV8 | Protein | https://doi.org/10.1371/journal.pone.0172819 |  |  |
| A0A287EIP9 | Genome | 10.1038/nature11543 |  |  |
| Q40053 | Protein | https://doi.org/10.1371/journal.pone.0172819 |  |  |
| A0A287FYP1 | Genome | 10.1038/nature11543 | D hordein | 96.99% |
| Q40054 | Protein | https://doi.org/10.1371/journal.pone.0172819 |  |  |
| I6TRS8 | Protein | https://doi.org/10.1371/journal.pone.0172819 |  |  |
| I6SW34 | Protein | https://doi.org/10.1371/journal.pone.0172819 |  |  |
| F2EA67 | Protein | https://doi.org/10.1371/journal.pone.0172819 |  |  |
| I6SW23 | Protein | https://doi.org/10.1371/journal.pone.0172819 |  |  |
| Q84LE9 | Protein | https://doi.org/10.1371/journal.pone.0172819 |  |  |
| M0XYT2 | Protein | 10.1021/acs.jproteome.5b00187 | γ1 hordeins | n/a |
| I6SJ17 | mRNA | 10.1186/1471-2229-12-184 | γ2 hordeins | 98.54% |
| >prf1604464A | Genomic translation | 10.1007/BF00039026 |  |  |
| P17990 | Protein | 10.1021/acs.jproteome.5b00187 |  |  |
| I6TMV6 | mRNA | 10.1186/1471-2229-12-184 |  |  |
| I6TEV2 | Protein | https://doi.org/10.1371/journal.pone.0172819 | γ3 hordeins | 96.11% |
| P80198 | Protein | 10.1371/journal.pone.0052139 https://doi.org/10.1371/journal.pone.0172819 |  |  |
| A0A287EEZ5 | Genome | 10.1038/nature11543 |  |  |
