# Supplementary material for: Mapping Coeliac Toxic Motifs in the Prolamin Seed Storage Proteins of Barley, Rye, and Oats Using a Curated Sequence Database
Source: Front Nutr. 2020 Jul 17;7:87. doi: 10.3389/fnut.2020.00087 (PMC7379453; doi:10.3389/fnut.2020.00087)
Supplement: Supplementary file 3 [file Table_3.DOCX]

**Table S3. UniProt accession number of all *S. cereale* sequences contained in the GluPro v 5.0 database.**

| **UniProt accession number** | **Evidence level** | **Supporting literature** | **GluPro classification** | **Sequence homology** |
| --- | --- | --- | --- | --- |
| H8Y0F9 | Genomic DNA | 10.1007/s10709-013-9729-2 | α-prolamin | n/a |
| C4NFN5 | cDNA | Conservation and evolution analysis of omega-secalin  genes from rye, triticale and 1BL.1RS translocation of wheat-rye | ω-secalin | 95.71% |
| C4NFN9 | Protein | https://doi.org/10.1371/journal.pone.0172819 |  |  |
| C4NFN8 | Protein | https://doi.org/10.1371/journal.pone.0172819 |  |  |
| C4NFN7 | cDNA | Conservation and evolution analysis of omega-secalin  genes from rye, triticale and 1BL.1RS translocation of wheat-rye |  |  |
| O04365 | cDNA | 10.1007/s004120050184 |  |  |
| Q05573 | Protein | https://doi.org/10.1371/journal.pone.0172819 |  |  |
| Q43639 | Protein | https://doi.org/10.1371/journal.pone.0172819 |  |  |
| C4NFN6 | cDNA | Conservation and evolution analysis of omega-secalin  genes from rye, triticale and 1BL.1RS translocation of wheat-rye |  |  |
| H8Y0K4 | Protein | https://doi.org/10.1371/journal.pone.0172819 | 40k γ-secalin | 79.18% |
| E5KZQ6 | cDNA | 10.1016/j.jcs.2012.05.013 |  |  |
| E5KZQ3 | Protein | https://doi.org/10.1371/journal.pone.0172819 |  |  |
| E5KZQ5 | Protein | https://doi.org/10.1371/journal.pone.0172819 |  |  |
| E5KZQ4 | Protein | https://doi.org/10.1371/journal.pone.0172819 |  |  |
| Q9FR41 | Protein | https://doi.org/10.1371/journal.pone.0172819 | 75k γ-secalin | 92.97% |
| K7WF86 | Protein | https://doi.org/10.1371/journal.pone.0172819 |  |  |
| E5KZQ2 | Protein | https://doi.org/10.1371/journal.pone.0172819 |  |  |
| A4GU92 | Protein | https://doi.org/10.1371/journal.pone.0172819 |  |  |
| A4GU93 | cDNA | 10.1016/j.jcs.2007.08.008 |  |  |
| K7WM28 | Protein | https://doi.org/10.1371/journal.pone.0172819 |  |  |
| K7WM32 | Protein | https://doi.org/10.1371/journal.pone.0172819 |  |  |
| K7WZB8 | Protein | https://doi.org/10.1371/journal.pone.0172819 |  |  |
| E5KZQ1 | Protein | https://doi.org/10.1371/journal.pone.0172819 |  |  |
| E5KZQ0 | Protein | https://doi.org/10.1371/journal.pone.0172819 |  |  |
| K7WJK0 | cDNA |  |  |  |
| K7XD33 | cDNA |  |  |  |
| E5KZP9 | Protein | https://doi.org/10.1371/journal.pone.0172819 |  |  |
| Q94IL6 | Protein | https://doi.org/10.1371/journal.pone.0172819 | y-type HMW secalin subunit | 87.78% |
| Q94IK8 | Protein | https://doi.org/10.1371/journal.pone.0172819 |  |  |
| Q94IL1 | cDNA | 10.1007/s00122-003-1234-z 10.1007/s001220100669 |  |  |
| Q93WM1 | Protein | https://doi.org/10.1371/journal.pone.0172819 |  |  |
| Q94IK7 | Protein | https://doi.org/10.1371/journal.pone.0172819 |  |  |
| D3XQB8 | cDNA |  |  |  |
| Q94IL4 | Protein | https://doi.org/10.1371/journal.pone.0172819 |  |  |
| Q94IL0 | Protein | https://doi.org/10.1371/journal.pone.0172819 | x-type HMW secalin subunit | 91.33% |
| D3XQB7 | Protein | https://doi.org/10.1371/journal.pone.0172819 |  |  |
| Q94IK9 | Protein | https://doi.org/10.1371/journal.pone.0172819 |  |  |
| Q93WF0 | Protein | https://doi.org/10.1371/journal.pone.0172819 |  |  |
| Q94IL2 | Protein | https://doi.org/10.1371/journal.pone.0172819 |  |  |
| Q94IL3 | cDNA | 10.1007/s00122-003-1234-z 10.1007/s001220100669 |  |  |
| Q94IK6 | cDNA | 10.1007/s00122-003-1234-z 10.1007/s001220100669 |  |  |
