# Supplementary material for: Mapping Coeliac Toxic Motifs in the Prolamin Seed Storage Proteins of Barley, Rye, and Oats Using a Curated Sequence Database
Source: Front Nutr. 2020 Jul 17;7:87. doi: 10.3389/fnut.2020.00087 (PMC7379453; doi:10.3389/fnut.2020.00087)
Supplement: Supplementary file 4 [file Table_4.DOCX]

**Table S4. UniProt accession number of all *A. sativa* sequences contained in the GluPro v 5.0 database**. The prolamin classification arising from sequence analysis is provided together with within-group sequence homology.

| **UniProt accession number** | **Evidence level** | **Supporting literature** | **GluPro Classification** | **Sequence homology** |
| --- | --- | --- | --- | --- |
| Q09071 | mRNA | 10.1105/tpc.1.9.913 | A | 89.67% |
| L0L5H3 | cDNA | 10.1371/journal.pone.0048365 |  |  |
| L0L8A0 | cDNA | 10.1371/journal.pone.0048365 |  |  |
| L0L8A4 | cDNA | 10.1371/journal.pone.0048365 |  |  |
| I4EP78 | cDNA | Avenins in oat (Avena sp.) do not contain the coeliac disease epitopes known from wheat, barley and rye |  |  |
| I4EP85 | cDNA | Avenins in oat (Avena sp.) do not contain the celiac disease epitopes known from wheat, barley and rye |  |  |
| L0L4J1 | cDNA | 10.1371/journal.pone.0048365 | B | 77.74% |
| Q38794 | cDNA | Analysis of seed storage protein genes of oats. |  |  |
| P80356 | Protein | 10.1105/tpc.1.9.913 https://doi.org/10.1371/journal.pone.0172819 |  |  |
| Q2EPY2 | cDNA | 10.20381/ruor-11456 |  |  |
| L0L6J5 | cDNA | 10.1371/journal.pone.0048365 |  |  |
| L0L5I0 | Protein | https://doi.org/10.1371/journal.pone.0172819 |  |  |
| I4EP88 | Protein | https://doi.org/10.1371/journal.pone.0172819 |  |  |
| L0L4I8 | Protein | https://doi.org/10.1016/j.foodchem.2018.11.110 |  |  |
| L0L6J0 | Protein | https://doi.org/10.1371/journal.pone.0172819 |  |  |
| L0L5H5 | Protein | https://doi.org/10.1371/journal.pone.0172819 | C | 89.30% |
| Q09072 | Protein | https://doi.org/10.1371/journal.pone.0172819 |  |  |
| L0L5G8 | Protein | https://doi.org/10.1371/journal.pone.0172819 |  |  |
| Q09114 | Protein | https://doi.org/10.1371/journal.pone.0172819 |  |  |
| L0L6K5 | Protein | https://doi.org/10.1016/j.foodchem.2018.11.110 |  |  |
| L0L841 | Protein | https://doi.org/10.1371/journal.pone.0172819 |  |  |
| L0L6K1 | Protein | https://doi.org/10.1371/journal.pone.0172819 |  |  |
| L0L8B6 | cDNA | 10.1371/journal.pone.0048365 |  |  |
