# Supplementary material for: Mapping Coeliac Toxic Motifs in the Prolamin Seed Storage Proteins of Barley, Rye, and Oats Using a Curated Sequence Database
Source: Front Nutr. 2020 Jul 17;7:87. doi: 10.3389/fnut.2020.00087 (PMC7379453; doi:10.3389/fnut.2020.00087)
Supplement: Supplementary file 5 [file Table_5.DOCX]

| **Origin Species**  **Supplementary Table S5. Coeliac toxic motif evaluation of sequences in the GluPro database family.**  Analysis was performed using Allergenonline database and tools to calculate the number of coeliac toxic motifs per sequence and average number of motifs per protein group. Density of coeliac toxic motifs was calculated by dividing the number of coeliac toxic motifs by the sequence length and the average ± standard deviation per protein group is also shown. Finally, the sequence coverage by coeliac toxic motifs was calculated using Protein Coverage Summarizer software (v1.3.6794). The average sequence coverage is also shown. | **Protein Classification** | **UniProt**  **accession**  **number** | **Sequence length** | **No. of coeliac toxic motifs** | | **Density of coeliac toxic motifs ± standard deviation** | | **Protein coverage %** | |
| --- | --- | --- | --- | --- | --- | --- | --- | --- | --- |
|  |  |  |  | **Individual** | **Average** | **Individual** | **Average** | **Individual** | **Average** |
| *T. aestivum*  (GluPro v1.2) | α-gliadin | A0A0K2QJY6 | 310 | 18 | 26.89 | 0.058 | 0.0902±  0.064 | 19.032 | 30.63 |
|  |  | A0A0K2QJC7 | 316 | 6 |  | 0.019 |  | 14.873 |  |
|  |  | A0A2R2Y441 | 313 | 8 |  | 0.026 |  | 28.115 |  |
|  |  | A0A1K0K0R0 | 290 | 40 |  | 0.138 |  | 27.241 |  |
|  |  | A0A0K2QJB4 | 293 | 36 |  | 0.123 |  | 41.98 |  |
|  |  | A0A1K0JNG6 | 308 | 65 |  | 0.211 |  | 42.857 |  |
|  |  | A0A0E3Z6M6 | 279 | 30 |  | 0.108 |  | 38.71 |  |
|  |  | A0A0K2QJX7 | 306 | 32 |  | 0.105 |  | 41.83 |  |
|  |  | A0A3B6PHC0 | 280 | 7 |  | 0.025 |  | 21.071 |  |
|  | δ-gliadin | A0A1D6RER0 | 317 | 0 | 0 | 0 | 0 | 0 | 0 |
|  |  | A0A2U8JD37 | 324 | 0 |  | 0 |  | 0 |  |
|  | Group I avenin-like | Q2A784 | 149 | 0 | 0 | 0 | 0 | 0 | 0 |
|  |  | P0CZ07 | 160 | 0 |  | 0 |  | 0 |  |
|  |  | P0CZ08 | 156 | 0 |  | 0 |  | 0 |  |
|  |  | D2KFH1 | 153 | 0 |  | 0 |  | 0 |  |
|  |  | P0CZ09 | 154 | 0 |  | 0 |  | 0 |  |
|  |  | P0CZ10 | 162 | 0 |  | 0 |  | 0 |  |
|  |  | P0CZ11 | 156 | 0 |  | 0 |  | 0 |  |
|  |  | A0A3B6RB11 | 153 | 0 |  | 0 |  | 0 |  |
|  |  | A0A3B6R7A6 | 155 | 0 |  | 0 |  | 0 |  |
|  |  | A0A3B6I2R2 | 152 | 0 |  | 0 |  | 0 |  |
|  |  | A0A3B6I494 | 161 | 0 |  | 0 |  | 0 |  |
|  |  | A0A3B6R7B8 | 157 | 0 |  | 0 |  | 0 |  |
|  |  | A0A3B6I296 | 157 | 0 |  | 0 |  | 0 |  |
|  |  | A0A3B5YQX6 | 206 | 0 |  | 0 |  | 0 |  |
|  | Group II avenin-like | G9I0S8 | 266 | 0 | 0 | 0 | 0 | 0 | 0 |
|  |  | G9I0S9 | 266 | 0 |  | 0 |  | 0 |  |
|  |  | G9I0T1 | 266 | 0 |  | 0 |  | 0 |  |
|  |  | G9I0T4 | 266 | 0 |  | 0 |  | 0 |  |
|  |  | G9I0T6 | 266 | 0 |  | 0 |  | 0 |  |
|  |  | G9I0T7 | 266 | 0 |  | 0 |  | 0 |  |
|  |  | G9I0U1 | 266 | 0 |  | 0 |  | 0 |  |
|  |  | G9I0U4 | 266 | 0 |  | 0 |  | 0 |  |
|  |  | G9I0U5 | 266 | 0 |  | 0 |  | 0 |  |
|  |  | V5M0W9 | 266 | 0 |  | 0 |  | 0 |  |
|  |  | V5M127 | 266 | 0 |  | 0 |  | 0 |  |
|  |  | V5M290 | 266 | 0 |  | 0 |  | 0 |  |
|  |  | V5M3L1 | 266 | 0 |  | 0 |  | 0 |  |
|  |  | V5M3L6 | 266 | 0 |  | 0 |  | 0 |  |
|  |  | A0A1W6C2L9 | 266 | 0 |  | 0 |  | 0 |  |
|  |  | A0A1W6C2M3 | 265 | 0 |  | 0 |  | 0 |  |
|  |  | A0A1W6C2L7 | 266 | 0 |  | 0 |  | 0 |  |
|  |  | A0A286QSW6 | 266 | 0 |  | 0 |  | 0 |  |
|  |  | A0A286QTK1 | 266 | 0 |  | 0 |  | 0 |  |
|  |  | A0A173DQY3 | 266 | 0 |  | 0 |  | 0 |  |
|  |  | A0A3G2CFH3 | 265 | 0 |  | 0 |  | 0 |  |
|  |  | A0A3B6I5M0 | 260 | 0 |  | 0 |  | 0 |  |
|  |  | A0A3B6T7X8 | 260 | 0 |  | 0 |  | 0 |  |
|  |  | A0A2H5BTG6 | 265 | 0 |  | 0 |  | 0 |  |
|  |  | A0A3G2CFA9 | 265 | 0 |  | 0 |  | 0 |  |
|  |  | A0A3B6RB62 | 260 | 0 |  | 0 |  | 0 |  |
|  |  | A0A3G2CFA8 | 266 | 0 |  | 0 |  | 0 |  |
|  |  | A0A3G2CFD4 | 264 | 0 |  | 0 |  | 0 |  |
|  |  | A0A173DQZ4 | 266 | 0 |  | 0 |  | 0 |  |
|  |  | A0A173DQZ7 | 266 | 0 |  | 0 |  | 0 |  |
|  |  | A0A173DR05 | 265 | 0 |  | 0 |  | 0 |  |
|  |  | A0A2H5BTG6 | 266 | 0 |  | 0 |  | 0 |  |
|  |  | Q2A783 | 267 | 0 |  | 0 |  | 0 |  |
|  |  | D6QZM8 | 266 | 0 |  | 0 |  | 0 |  |
|  |  | B8YG97 | 266 | 0 |  | 0 |  | 0 |  |
|  |  | P0CZ05 | 267 | 0 |  | 0 |  | 0 |  |
|  |  | P0CZ06 | 266 | 0 |  | 0 |  | 0 |  |
|  |  | A5A4L5 | 266 | 0 |  | 0 |  | 0 |  |
|  |  | A7XUQ5 | 266 | 0 |  | 0 |  | 0 |  |
|  |  | A5A4L4 | 266 | 0 |  | 0 |  | 0 |  |
|  |  | D0EWS4 | 266 | 0 |  | 0 |  | 0 |  |
|  |  | D6QZM5 | 266 | 0 |  | 0 |  | 0 |  |
|  |  | D6QZM4 | 266 | 0 |  | 0 |  | 0 |  |
| *T. turgidum* ssp durum  (GluPro v 2.0) | α-gliadin | 182939 | 231 | 15 | 14.93 | 0.064935 | 0.055±  0.034 | 22.944 | 28.96 |
|  |  | 183774 | 277 | 7 |  | 0.025271 |  | 20.486 |  |
|  |  | 183775 | 216 | 7 |  | 0.032407 |  | 21.3 |  |
|  |  | 183776 | 249 | 7 |  | 0.028112 |  | 12.963 |  |
|  |  | 183778 | 260 | 7 |  | 0.026923 |  | 23.695 |  |
|  |  | 183924 | 289 | 20 |  | 0.069204 |  | 32.692 |  |
|  |  | 183925 | 264 | 33 |  | 0.125 |  | 44.291 |  |
|  |  | 183926 | 264 | 32 |  | 0.121212 |  | 48.485 |  |
|  |  | 187277 | 197 | 5 |  | 0.025381 |  | 18.782 |  |
|  |  | 187279 | 250 | 13 |  | 0.052 |  | 33.6 |  |
|  |  | A0A0E3UQW2 | 287 | 19 |  | 0.066202 |  | 27.875 |  |
|  |  | A0A0E3UQW4 | 289 | 32 |  | 0.110727 |  | 44.291 |  |
|  |  | A0A0E3UQW5 | 256 | 11 |  | 0.042969 |  | 35.938 |  |
|  |  | A0A0E3UR92 | 213 | 7 |  | 0.032864 |  | 33.803 |  |
|  |  | A0A0E3UR95 | 290 | 22 |  | 0.075862 |  | 33.793 |  |
|  |  | A0A0E3UR97 | 285 | 32 |  | 0.112281 |  | 44.912 |  |
|  |  | A0A0E3UR98 | 285 | 27 |  | 0.094737 |  | 43.86 |  |
|  |  | A0A0E3Z552 | 315 | 6 |  | 0.019048 |  | 14.921 |  |
|  |  | A0A0E3Z559 | 285 | 29 |  | 0.101754 |  | 41.404 |  |
|  |  | A0A0E3Z563 | 287 | 23 |  | 0.080139 |  | 43.206 |  |
|  |  | A0A0E3Z5B3 | 289 | 15 |  | 0.051903 |  | 23.183 |  |
|  |  | A0A0E3Z5B6 | 285 | 32 |  | 0.112281 |  | 44.912 |  |
|  |  | A0A0E3Z5Z1 | 285 | 21 |  | 0.073684 |  | 39.649 |  |
|  |  | A0A0E3Z5Z6 | 285 | 20 |  | 0.070175 |  | 29.825 |  |
|  |  | A0A0E3Z6Q1 | 287 | 22 |  | 0.076655 |  | 34.843 |  |
|  |  | A0A0E3Z6Q7 | 287 | 28 |  | 0.097561 |  | 41.115 |  |
|  |  | A0A0E3Z7J9 | 313 | 7 |  | 0.022364 |  | 23.003 |  |
|  |  | A0A0E3Z7K2 | 286 | 19 |  | 0.066434 |  | 29.021 |  |
|  |  | A0A446V2H3 | 247 | 7 |  | 0.02834 |  | 23.077 |  |
|  |  | A0A446V2H9 | 245 | 7 |  | 0.028571 |  | 23.265 |  |
|  |  | A0A446V2I3 | 253 | 13 |  | 0.051383 |  | 33.992 |  |
|  |  | A0A446V2I9 | 247 | 9 |  | 0.036437 |  | 32.389 |  |
|  |  | A0A446V2J2 | 282 | 22 |  | 0.078014 |  | 34.752 |  |
|  |  | A0A446V2J5 | 285 | 32 |  | 0.112281 |  | 44.912 |  |
|  |  | A0A446V2J7 | 196 | 14 |  | 0.071429 |  | 18.367 |  |
|  |  | A0A446V2J8 | 245 | 27 |  | 0.110204 |  | 44.082 |  |
|  |  | A0A446V2J9 | 220 | 31 |  | 0.140909 |  | 53.636 |  |
|  |  | A0A446V2K2 | 295 | 32 |  | 0.108475 |  | 43.39 |  |
|  |  | A0A446V2K5 | 290 | 31 |  | 0.106897 |  | 42.414 |  |
|  |  | A0A446V2K7 | 233 | 16 |  | 0.06867 |  | 28.755 |  |
|  |  | A0A446V2K9 | 262 | 32 |  | 0.122137 |  | 48.855 |  |
|  |  | A0A446V2L4 | 223 | 26 |  | 0.116592 |  | 52.018 |  |
|  |  | A0A446V2L5 | 256 | 20 |  | 0.078125 |  | 33.203 |  |
|  |  | A0A446V2L7 | 246 | 7 |  | 0.028455 |  | 23.171 |  |
|  |  | A0A446V2L8 | 287 | 24 |  | 0.083624 |  | 41.463 |  |
|  |  | A0A446V2M4 | 257 | 24 |  | 0.093385 |  | 46.304 |  |
|  |  | A0A446V2M7 | 188 | 28 |  | 0.148936 |  | 60.638 |  |
|  |  | A0A446V2M9 | 291 | 25 |  | 0.085911 |  | 41.581 |  |
|  |  | A0A446V2N5 | 245 | 31 |  | 0.126531 |  | 48.163 |  |
|  |  | A0A446V2P0 | 288 | 31 |  | 0.107639 |  | 44.444 |  |
|  |  | A0A446V2P5 | 245 | 19 |  | 0.077551 |  | 28.92 |  |
|  |  | A0A446V2Q0 | 257 | 9 |  | 0.035019 |  | 28.016 |  |
|  |  | A0A446V2Q9 | 245 | 10 |  | 0.040816 |  | 34.694 |  |
|  |  | A0A446W085 | 298 | 7 |  | 0.02349 |  | 24.161 |  |
|  |  | A0A446W087 | 299 | 7 |  | 0.023411 |  | 24.08 |  |
|  |  | A0A446W089 | 280 | 18 |  | 0.064286 |  | 26.786 |  |
|  |  | A0A446W093 | 325 | 7 |  | 0.021538 |  | 22.154 |  |
|  |  | A0A446W094 | 314 | 7 |  | 0.022293 |  | 22.93 |  |
|  |  | A0A446W095 | 291 | 7 |  | 0.024055 |  | 24.742 |  |
|  |  | A0A446W096 | 310 | 7 |  | 0.022581 |  | 19.032 |  |
|  |  | A0A446W099 | 298 | 14 |  | 0.04698 |  | 25.503 |  |
|  |  | A0A446W0A1 | 291 | 8 |  | 0.027491 |  | 24.742 |  |
|  |  | A0A446W0A3 | 299 | 7 |  | 0.023411 |  | 24.08 |  |
|  |  | A0A446W0A5 | 319 | 8 |  | 0.025078 |  | 27.586 |  |
|  |  | A0A446W0A6 | 289 | 7 |  | 0.024221 |  | 24.913 |  |
|  |  | A0A446W0A9 | 310 | 7 |  | 0.022581 |  | 23.226 |  |
|  |  | A0A446W0B2 | 320 | 3 |  | 0.009375 |  | 7.812 |  |
|  |  | A0A446W0B4 | 253 | 18 |  | 0.071146 |  | 33.202 |  |
|  |  | A0A446W0B5 | 354 | 14 |  | 0.039548 |  | 28.814 |  |
|  |  | A0A446W0B7 | 309 | 7 |  | 0.022654 |  | 19.094 |  |
|  |  | A0A446W0B9 | 287 | 7 |  | 0.02439 |  | 25.087 |  |
|  |  | A0A446W0C2 | 303 | 14 |  | 0.046205 |  | 22.112 |  |
|  |  | A0A446W0C7 | 306 | 3 |  | 0.009804 |  | 8.17 |  |
|  |  | A0A446W0C8 | 268 | 7 |  | 0.026119 |  | 16.045 |  |
|  |  | A0A446W0C9 | 291 | 7 |  | 0.024055 |  | 20.275 |  |
|  |  | A0A446W0D4 | 291 | 7 |  | 0.024055 |  | 20.275 |  |
|  |  | A0A446W0D7 | 292 | 19 |  | 0.065068 |  | 28.767 |  |
|  |  | A0A446W0D8 | 292 | 17 |  | 0.058219 |  | 20.89 |  |
|  |  | A0A446W0E7 | 292 | 7 |  | 0.023973 |  | 24.658 |  |
|  |  | A0A446W0E8 | 308 | 7 |  | 0.022727 |  | 23.701 |  |
|  |  | A0A446W0F5 | 300 | 7 |  | 0.023333 |  | 24 |  |
|  |  | A0A446W0F7 | 280 | 7 |  | 0.025 |  | 21.071 |  |
|  |  | A0A446W0G6 | 306 | 7 |  | 0.022876 |  | 23.529 |  |
|  |  | A0A446W0H6 | 195 | 7 |  | 0.035897 |  | 36.923 |  |
|  |  | A0A446W0I4 | 292 | 7 |  | 0.023973 |  | 20.205 |  |
|  |  | A0A446W0J7 | 317 | 14 |  | 0.044164 |  | 23.975 |  |
|  |  | A0A446W0K7 | 293 | 7 |  | 0.023891 |  | 24.573 |  |
|  |  | A0A446W0L8 | 204 | 7 |  | 0.034314 |  | 35.294 |  |
|  |  | A0A446W0N0 | 294 | 7 |  | 0.02381 |  | 20.068 |  |
|  |  | A0A446W1C0 | 302 | 16 |  | 0.05298 |  | 18.874 |  |
|  |  | A0A446W1C2 | 306 | 16 |  | 0.052288 |  | 18.627 |  |
|  |  | A0A446W1C4 | 280 | 16 |  | 0.057143 |  | 20.357 |  |
|  |  | A0A446W1D6 | 282 | 16 |  | 0.056738 |  | 20.213 |  |
|  |  | A0A446W1F2 | 282 | 16 |  | 0.056738 |  | 20.213 |  |
|  |  | A0A446W1F6 | 262 | 15 |  | 0.057252 |  | 19.847 |  |
|  |  | A0A446W1H7 | 280 | 16 |  | 0.057143 |  | 20.357 |  |
|  |  | A0A446W1J4 | 306 | 16 |  | 0.052288 |  | 18.627 |  |
|  |  | D2X6C8 | 275 | 12 |  | 0.043636 |  | 28.727 |  |
|  |  | D2X6C9 | 300 | 24 |  | 0.08 |  | 25.333 |  |
|  |  | D2X6D0 | 288 | 18 |  | 0.0625 |  | 27.083 |  |
|  |  | D2X6D1 | 313 | 8 |  | 0.025559 |  | 28.115 |  |
|  |  | D2X6D2 | 277 | 14 |  | 0.050542 |  | 24.549 |  |
|  |  | D2X6D3 | 320 | 8 |  | 0.025 |  | 27.5 |  |
|  |  | D2X6D4 | 281 | 2 |  | 0.007117 |  | 12.456 |  |
|  |  | D2X6D5 | 302 | 1 |  | 0.003311 |  | 6.623 |  |
|  |  | D2X6D8 | 288 | 4 |  | 0.013889 |  | 18.056 |  |
|  |  | D2X6D9 | 313 | 8 |  | 0.025559 |  | 28.115 |  |
|  |  | Q2V5Z6 | 287 | 22 |  | 0.076655 |  | 34.843 |  |
|  |  | Q2V5Z7 | 314 | 6 |  | 0.019108 |  | 21.019 |  |
|  |  | Q5NDA5 | 286 | 25 |  | 0.087413 |  | 39.86 |  |
|  | Group I avenin-like | 182970 | 181 | 0 | 0 | 0 | 0 | 0 | 0 |
|  |  | 185636 | 172 | 0 |  | 0 |  | 0 |  |
|  |  | 186240 | 168 | 0 |  | 0 |  | 0 |  |
|  |  | 186241 | 152 | 0 |  | 0 |  | 0 |  |
|  |  | 186242 | 150 | 0 |  | 0 |  | 0 |  |
|  |  | A0A446RKG6 | 128 | 0 |  | 0 |  | 0 |  |
|  |  | A0A446RKK3 | 156 | 0 |  | 0 |  | 0 |  |
|  |  | A0A446RL10 | 168 | 0 |  | 0 |  | 0 |  |
|  |  | A0A446WXR1 | 173 | 0 |  | 0 |  | 0 |  |
|  |  | A0A446WXR7 | 175 | 0 |  | 0 |  | 0 |  |
|  |  | A0A446WXS8 | 173 | 0 |  | 0 |  | 0 |  |
|  |  | A0A446WXT0 | 173 | 0 |  | 0 |  | 0 |  |
|  |  | A0A446WXT3 | 173 | 0 |  | 0 |  | 0 |  |
|  |  | A0A446WXT5 | 173 | 0 |  | 0 |  | 0 |  |
|  |  | A0A446WXV0 | 173 | 0 |  | 0 |  | 0 |  |
|  |  | A0A446WXW6 | 157 | 0 |  | 0 |  | 0 |  |
|  | Group II avenin-like | 184653 | 167 | 0 | 0 | 0 | 0 | 0 | 0 |
|  |  | 184654 | 285 | 0 |  | 0 |  | 0 |  |
|  |  | 184655 | 167 | 0 |  | 0 |  | 0 |  |
|  |  | 184656 | 233 | 0 |  | 0 |  | 0 |  |
|  |  | 188204 | 280 | 0 |  | 0 |  | 0 |  |
|  |  | A0A446WXS7 | 284 | 0 |  | 0 |  | 0 |  |
|  | δ-gliadin | A0A446IHB0 | 219 | 0 | 0 | 0 | 0 | 0 | 0 |
|  |  | A0A446IHE0 | 212 | 0 |  | 0 |  | 0 |  |
|  | γ-gliadin | A0A446IHB5 | 255 | 6 | 21.72 | 0.023529 | 0.074±0.0325 | 12.549 | 35.57 |
|  |  | A0A446IHB8 | 280 | 15 |  | 0.053571 |  | 31.071 |  |
|  |  | A0A446IHC0 | 283 | 15 |  | 0.053004 |  | 22.968 |  |
|  |  | A0A446IHC1 | 315 | 17 |  | 0.053968 |  | 34.921 |  |
|  |  | A0A446IHC3 | 315 | 24 |  | 0.07619 |  | 35.294 |  |
|  |  | A0A446IHC5 | 315 | 18 |  | 0.057143 |  | 40.952 |  |
|  |  | A0A446IHC7 | 251 | 11 |  | 0.043825 |  | 17.131 |  |
|  |  | A0A446IHD2 | 285 | 18 |  | 0.063158 |  | 36.14 |  |
|  |  | A0A446IHD5 | 285 | 18 |  | 0.063158 |  | 34.737 |  |
|  |  | A0A446IHD6 | 200 | 5 |  | 0.025 |  | 11.5 |  |
|  |  | A0A446IHD7 | 331 | 22 |  | 0.066465 |  | 42.598 |  |
|  |  | A0A446IHE6 | 300 | 18 |  | 0.06 |  | 33 |  |
|  |  | A0A446IHF4 | 292 | 23 |  | 0.078767 |  | 35.959 |  |
|  |  | A0A446IHJ1 | 285 | 18 |  | 0.063158 |  | 34.737 |  |
|  |  | A0A446IHK0 | 282 | 21 |  | 0.074468 |  | 37.589 |  |
|  |  | A0A446JG43 | 302 | 42 |  | 0.139073 |  | 59.603 |  |
|  |  | A0A446JG55 | 291 | 22 |  | 0.075601 |  | 26.117 |  |
|  |  | A0A446JG61 | 302 | 42 |  | 0.139073 |  | 66.225 |  |
|  |  | A0A446JG74 | 310 | 22 |  | 0.070968 |  | 39.032 |  |
|  |  | A0A446JG83 | 291 | 20 |  | 0.068729 |  | 19.244 |  |
|  |  | A0A446JG84 | 302 | 41 |  | 0.135762 |  | 59.603 |  |
|  |  | A0A446JG85 | 311 | 20 |  | 0.064309 |  | 38.585 |  |
|  |  | A0A446JG88 | 291 | 21 |  | 0.072165 |  | 23.368 |  |
|  |  | A0A446JG98 | 326 | 17 |  | 0.052147 |  | 33.742 |  |
|  |  | A0A446JG99 | 291 | 20 |  | 0.068729 |  | 19.244 |  |
|  |  | A0A446JGA9 | 331 | 17 |  | 0.05136 |  | 33.233 |  |
|  |  | A0A446JGE5 | 331 | 17 |  | 0.05136 |  | 33.233 |  |
|  |  | A0A446JGF8 | 297 | 22 |  | 0.074074 |  | 41.751 |  |
|  |  | A0A446JGQ5 | 331 | 17 |  | 0.05136 |  | 33.233 |  |
|  |  | A0A446JGQ8 | 282 | 37 |  | 0.131206 |  | 59.929 |  |
|  |  | Q41602 | 280 | 42 |  | 0.15 |  | 71.429 |  |
|  |  | Q6EEW3 | 242 | 14 |  | 0.057851 |  | 20.661 |  |
|  |  | Q6EEW4 | 275 | 23 |  | 0.083636 |  | 30.909 |  |
|  |  | Q6EEW5 | 239 | 16 |  | 0.066946 |  | 21.339 |  |
|  |  | Q6EEW6 | 275 | 20 |  | 0.072727 |  | 20.364 |  |
|  |  | Q84M19 | 282 | 41 |  | 0.14539 |  | 68.44 |  |
|  | HMW glutenin subunit | A0A0E4G9A4 | 717 | 13 | 14.43 | 0.018131 | 0.019±0.007 | 8.787 | 15.46 |
|  |  | A0A2L1K3K6 | 686 | 14 |  | 0.020408 |  | 11.37 |  |
|  |  | K4N1X7 | 795 | 15 |  | 0.018868 |  | 22.642 |  |
|  |  | Q6UJY5 | 824 | 16 |  | 0.019417 |  | 19.053 |  |
|  |  | Q6UJY7 | 720 | 14 |  | 0.019444 |  | 10.833 |  |
|  |  | Q84TG6 | 720 | 14 |  | 0.019444 |  | 12.917 |  |
|  |  | Q8RVX0 | 795 | 15 |  | 0.018868 |  | 22.642 |  |
|  | LMW glutenin | CAA36063.1 | 295 | 1 | 3.36 | 0.00339 | 0.011±0.011 | 3.051 | 8.54 |
|  |  | 185860 | 229 | 0 |  | 0 |  | 0 |  |
|  |  | 185861 | 336 | 2 |  | 0.005952 |  | 4.464 |  |
|  |  | 185862 | 175 | 0 |  | 0 |  | 0 |  |
|  |  | 185863 | 345 | 3 |  | 0.008696 |  | 6.957 |  |
|  |  | 185864 | 337 | 2 |  | 0.005935 |  | 4.451 |  |
|  |  | 185866 | 292 | 1 |  | 0.003425 |  | 3.082 |  |
|  |  | 185867 | 250 | 0 |  | 0 |  | 0 |  |
|  |  | 185868 | 294 | 2 |  | 0.006803 |  | 5.102 |  |
|  |  | A0A2P1BXV0 | 350 | 2 |  | 0.005714 |  | 4.286 |  |
|  |  | A0A446IHD8 | 258 | 9 |  | 0.034884 |  | 15.504 |  |
|  |  | A0A446IHD9 | 309 | 9 |  | 0.029126 |  | 17.799 |  |
|  |  | A0A446IHE3 | 242 | 9 |  | 0.03719 |  | 10.744 |  |
|  |  | A0A446IHF1 | 259 | 1 |  | 0.003861 |  | 5.792 |  |
|  |  | A0A446IHF7 | 280 | 9 |  | 0.032143 |  | 19.643 |  |
|  |  | A0A446IHH8 | 283 | 1 |  | 0.003534 |  | 4.24 |  |
|  |  | A0A446IHK2 | 257 | 0 |  | 0 |  | 0 |  |
|  |  | A0A446IHK8 | 253 | 9 |  | 0.035573 |  | 16.206 |  |
|  |  | A0A446IHQ7 | 291 | 1 |  | 0.003436 |  | 4.124 |  |
|  |  | A7XDG0 | 363 | 3 |  | 0.008264 |  | 12.948 |  |
|  |  | D5FPE1 | 392 | 3 |  | 0.007653 |  | 14.286 |  |
|  |  | D5FPE4 | 350 | 2 |  | 0.005714 |  | 4.286 |  |
|  |  | G9AYK6 | 388 | 9 |  | 0.023196 |  | 18.041 |  |
|  |  | O49958 | 350 | 4 |  | 0.011429 |  | 8.571 |  |
|  |  | Q0Q2J0 | 297 | 3 |  | 0.010101 |  | 7.744 |  |
|  |  | Q0Q2J1 | 334 | 3 |  | 0.008982 |  | 11.377 |  |
|  |  | Q41603 | 285 | 1 |  | 0.003509 |  | 4.211 |  |
|  |  | Q68VI0 | 381 | 2 |  | 0.005249 |  | 7.874 |  |
|  |  | Q84NE4 | 388 | 4 |  | 0.010309 |  | 17.01 |  |
|  |  | Q9FEQ1 | 301 | 1 |  | 0.003322 |  | 3.987 |  |
|  |  | Q9FEQ2 | 387 | 9 |  | 0.023256 |  | 18.088 |  |
| *H. vulgare*  (GluPro v 3.0) | Group I avenin-like | A0A287JK04 | 143 | 0 | 0 | 0.000 | 0 | 0 | 0 |
|  |  | M0V4S8 | 159 | 0 |  | 0.000 |  | 0 |  |
|  |  | M0VEH1 | 164 | 0 |  | 0.000 |  | 0 |  |
|  |  | F2EGD5 | 173 | 0 |  | 0.000 |  | 0 |  |
|  |  | M0VH55 | 173 | 0 |  | 0.000 |  | 0 |  |
|  | Group II avenin-like | A7XUQ7 | 284 | 0 | 0 | 0.000 | 0 | 0 | 0 |
|  |  | M0VWJ3 | 314 | 0 |  | 0.000 |  | 0 |  |
|  | B1 hordein | I6TMW0 | 253 | 3 | 6.24 | 0.012 | 0.022±0.008 | 10.672 | 13.47 |
|  |  | I6QM99 | 260 | 0 |  | 0.000 |  | 0 |  |
|  |  | I6SW25 | 267 | 6 |  | 0.022 |  | 8.614 |  |
|  |  | Q40021 | 271 | 5 |  | 0.018 |  | 8.856 |  |
|  |  | I6R4A7 | 271 | 5 |  | 0.018 |  | 8.856 |  |
|  |  | I6TRT2 | 278 | 3 |  | 0.011 |  | 10.791 |  |
|  |  | I6SJ22 | 286 | 3 |  | 0.010 |  | 12.238 |  |
|  |  | Q40026 | 290 | 9 |  | 0.031 |  | 14.828 |  |
|  |  | Q3LTR1 | 290 | 7 |  | 0.024 |  | 17.931 |  |
|  |  | I6TMV2 | 290 | 9 |  | 0.031 |  | 14.828 |  |
|  |  | P06470 | 293 | 7 |  | 0.024 |  | 17.747 |  |
|  |  | A0A287EFE1 | 293 | 7 |  | 0.024 |  | 17.747 |  |
|  |  | I6SJ13 | 294 | 9 |  | 0.031 |  | 14.626 |  |
|  |  | A0A287EFF7 | 294 | 6 |  | 0.020 |  | 14.286 |  |
|  |  | A0A287EFH8 | 296 | 8 |  | 0.027 |  | 14.865 |  |
|  |  | Q2XQF0 | 297 | 9 |  | 0.030 |  | 17.845 |  |
|  |  | A0A287EFD4 | 297 | 8 |  | 0.027 |  | 14.815 |  |
|  |  | A0A287EFB3 | 297 | 9 |  | 0.030 |  | 17.845 |  |
|  |  | A0A287EFG2 | 298 | 4 |  | 0.013 |  | 12.416 |  |
|  |  | A0A0K2GRS6 | 298 | 7 |  | 0.023 |  | 15.436 |  |
|  |  | Q3YAF9 | 300 | 7 |  | 0.023 |  | 17.667 |  |
|  | B3 hordein | A0A287EIW5 | 303 | 7 | 5.2 | 0.023 | 0.018±0.008 | 8.911 | 11.04 |
|  |  | Q0PIV6 | 290 | 8 |  | 0.028 |  | 16.552 |  |
|  |  | A0A287EIZ3 | 286 | 7 |  | 0.024 |  | 9.441 |  |
|  |  | I6SW30 | 284 | 6 |  | 0.021 |  | 13.732 |  |
|  |  | I6TMW4 | 277 | 7 |  | 0.025 |  | 12.635 |  |
|  |  | I6SJ26 | 310 | 7 |  | 0.023 |  | 19.677 |  |
|  |  | I6TRT5 | 279 | 7 |  | 0.025 |  | 11.828 |  |
|  |  | I6TEV5 | 310 | 7 |  | 0.023 |  | 19.677 |  |
|  |  | A0A287EJ06 | 260 | 7 |  | 0.027 |  | 10.385 |  |
|  |  | C7FB15 | 261 | 2 |  | 0.008 |  | 6.897 |  |
|  |  | C7FB13 | 265 | 2 |  | 0.008 |  | 6.792 |  |
|  |  | C7FB14 | 265 | 2 |  | 0.008 |  | 6.792 |  |
|  |  | Q2XQF1 | 265 | 2 |  | 0.008 |  | 6.792 |  |
|  |  | C7FB16 | 265 | 5 |  | 0.019 |  | 8.679 |  |
|  |  | C7FB17 | 265 | 2 |  | 0.008 |  | 6.792 |  |
|  | C hordein | A0A287EIM7 | 445 | 35 | 22.17 | 0.079 | 0.066±0.025 | 53.483 | 40.01 |
|  |  | A0A287EEX5 | 452 | 19 |  | 0.042 |  | 31.195 |  |
|  |  | Q41210 | 310 | 35 |  | 0.113 |  | 59.032 |  |
|  |  | I6TEV8 | 302 | 13 |  | 0.043 |  | 33.113 |  |
|  |  | A0A287EIP9 | 249 | 18 |  | 0.072 |  | 30.924 |  |
|  |  | Q40053 | 260 | 13 |  | 0.050 |  | 32.308 |  |
|  | D hordein | A0A287FYP1 | 625 | 0 | 0 | 0.000 | 0 | 0 | 0 |
|  |  | Q40054 | 707 | 0 |  | 0.000 |  | 0 |  |
|  |  | I6TRS8 | 727 | 0 |  | 0.000 |  | 0 |  |
|  |  | I6SW34 | 747 | 0 |  | 0.000 |  | 0 |  |
|  |  | F2EA67 | 747 | 0 |  | 0.000 |  | 0 |  |
|  |  | I6SW23 | 747 | 0 |  | 0.000 |  | 0 |  |
|  |  | Q84LE9 | 757 | 0 |  | 0.000 |  | 0 |  |
|  | γ1 hordein | M0XYT2 | 343 | 3 | 3 | 0.009 | 0.009±0 | 4.373 | 4.37 |
|  | γ2 hordein | I6SJ17 | 305 | 12 | 11 | 0.039 | 0.036±0.006 | 7.541 | 6.89 |
|  |  | 1604464A | 305 | 12 |  | 0.039 |  | 7.541 |  |
|  |  | P17990 | 305 | 12 |  | 0.039 |  | 7.541 |  |
|  |  | I6TMV6 | 305 | 8 |  | 0.026 |  | 4.918 |  |
|  | γ3 hordein | I6TEV2 | 295 | 1 | 1 | 0.003 | 0.003±0 | 3.051 | 3.00 |
|  |  | P80198 | 289 | 1 |  | 0.003 |  | 3.114 |  |
|  |  | A0A287EEZ5 | 319 | 1 |  | 0.003 |  | 2.821 |  |
| *S. cereal*  (GluPro v 4.0) | α-type prolamin | H8Y0F9 | 282 | 1 | 1 | 0.004 | 0.004±0 | 3.191 | 3.19 |
|  | ω-secalin | C4NFN5 | 357 | 33 | 35.38 | 0.092 | 0.099±0.008 | 43.697 | 52.87 |
|  |  | C4NFN9 | 357 | 34 |  | 0.095 |  | 54.062 |  |
|  |  | C4NFN8 | 357 | 39 |  | 0.109 |  | 54.622 |  |
|  |  | C4NFN7 | 357 | 38 |  | 0.106 |  | 48.739 |  |
|  |  | O04365 | 357 | 39 |  | 0.109 |  | 63.025 |  |
|  |  | Q05573 | 357 | 32 |  | 0.090 |  | 55.182 |  |
|  |  | Q43639 | 357 | 33 |  | 0.092 |  | 56.303 |  |
|  |  | C4NFN6 | 357 | 35 |  | 0.098 |  | 47.339 |  |
|  | 40k γ-secalin | H8Y0K4 | 210 | 2 | 4 | 0.010 | 0.017±0.017 | 9.524 | 12.37 |
|  |  | E5KZQ6 | 202 | 0 |  | 0.000 |  | 12.195 |  |
|  |  | E5KZQ4 | 247 | 10 |  | 0.040 |  | 15.385 |  |
|  | 75k γ-secalin | Q9FR41 | 455 | 34 | 28.2 | 0.075 | 0.063±0.018 | 36.044 | 31.37 |
|  |  | E5KZQ5 | 246 | 4 |  | 0.016 |  | 12.195 |  |
|  |  | K7WF86 | 404 | 14 |  | 0.035 |  | 25.99 |  |
|  |  | E5KZQ2 | 407 | 32 |  | 0.079 |  | 31.941 |  |
|  |  | A4GU92 | 455 | 34 |  | 0.075 |  | 35.385 |  |
|  |  | E5KZQ3 | 314 | 12 |  | 0.038 |  | 17.197 |  |
|  |  | A4GU93 | 455 | 32 |  | 0.070 |  | 34.066 |  |
|  |  | K7WM28 | 464 | 27 |  | 0.058 |  | 31.897 |  |
|  |  | K7WM32 | 464 | 27 |  | 0.058 |  | 31.897 |  |
|  |  | K7WZB8 | 471 | 33 |  | 0.070 |  | 33.97 |  |
|  |  | E5KZQ1 | 471 | 34 |  | 0.072 |  | 36.73 |  |
|  |  | E5KZQ0 | 477 | 35 |  | 0.073 |  | 35.849 |  |
|  |  | K7WJK0 | 477 | 35 |  | 0.073 |  | 35.849 |  |
|  |  | K7XD33 | 477 | 35 |  | 0.073 |  | 35.849 |  |
|  |  | E5KZP9 | 479 | 35 |  | 0.073 |  | 35.699 |  |
|  | y-type HMW secalin subunit | Q94IL6 | 707 | 2 | 2 | 0.003 | 0.003±0 | 8.487 | 7.45 |
|  |  | Q94IK8 | 713 | 2 |  | 0.003 |  | 8.415 |  |
|  |  | Q94IL1 | 713 | 2 |  | 0.003 |  | 8.415 |  |
|  |  | Q93WM1 | 713 | 2 |  | 0.003 |  | 8.415 |  |
|  |  | Q94IK7 | 713 | 2 |  | 0.003 |  | 8.415 |  |
|  |  | D3XQB8 | 760 | 2 |  | 0.003 |  | 5.921 |  |
|  |  | Q94IL4 | 737 | 2 |  | 0.003 |  | 4.071 |  |
|  | x-type HMW secalin subunit | Q94IL0 | 743 | 12 | 9.14 | 0.016 | 0.012±0.006 | 16.016 | 14.35 |
|  |  | D3XQB7 | 754 | 13 |  | 0.017 |  | 14.324 |  |
|  |  | Q94IK9 | 754 | 4 |  | 0.005 |  | 11.936 |  |
|  |  | Q93WF0 | 754 | 13 |  | 0.017 |  | 15.782 |  |
|  |  | Q94IL2 | 754 | 13 |  | 0.017 |  | 13.793 |  |
|  |  | Q94IL3 | 766 | 4 |  | 0.005 |  | 15.144 |  |
|  |  | Q94IK6 | 781 | 5 |  | 0.006 |  | 13.444 |  |
| 1. *sativa*   (GluPro v 5.0) | Avenin A | Q09071 | 181 | 1 | 0.8 | 0.006 | 0.0043±0.0023 | 8.84 | 6.28 |
|  |  | L0L5H3 | 181 | 1 |  | 0.006 |  | 8.84 |  |
|  |  | L0L6J7 | 181 | 1 |  | 0.006 |  | 8.84 |  |
|  |  | L0L833 | 189 | 1 |  | 0.005 |  | 7.407 |  |
|  |  | L0L8A0 | 188 | 1 |  | 0.005 |  | 7.447 |  |
|  |  | L0L837 | 189 | 1 |  | 0.005 |  | 7.407 |  |
|  |  | L0L8A4 | 189 | 1 |  | 0.005 |  | 7.407 |  |
|  |  | I4EP78 | 212 | 1 |  | 0.005 |  | 6.604 |  |
|  |  | I4EP86 | 209 | 0 |  | 0.000 |  | 0 |  |
|  |  | I4EP85 | 209 | 0 |  | 0.000 |  | 0 |  |
|  | Avenin B | L0L4J1 | 209 | 1 | 1.67 | 0.005 | 0.0069±0.0068 | 7.656 | 8.42 |
|  |  | Q38794 | 209 | 1 |  | 0.005 |  | 7.656 |  |
|  |  | P80356 | 220 | 1 |  | 0.005 |  | 7.273 |  |
|  |  | Q2EPY2 | 212 | 1 |  | 0.005 |  | 7.547 |  |
|  |  | L0L6J5 | 211 | 1 |  | 0.005 |  | 7.583 |  |
|  |  | L0L5I0 | 229 | 1 |  | 0.004 |  | 6.987 |  |
|  |  | I4EP88 | 242 | 1 |  | 0.004 |  | 3.719 |  |
|  |  | L0L4I8 | 250 | 1 |  | 0.004 |  | 6.4 |  |
|  |  | L0L6J0 | 281 | 7 |  | 0.025 |  | 20.996 |  |
|  | Avenin C | L0L5H5 | 214 | 5 | 6.88 | 0.023 | 0.0336±0.011 | 17.757 | 17.15 |
|  |  | Q09072 | 222 | 8 |  | 0.036 |  | 17.568 |  |
|  |  | L0L5G8 | 223 | 5 |  | 0.022 |  | 17.04 |  |
|  |  | Q09114 | 182 | 9 |  | 0.049 |  | 20.879 |  |
|  |  | L0L6K5 | 201 | 9 |  | 0.045 |  | 18.905 |  |
|  |  | L0L841 | 200 | 8 |  | 0.040 |  | 19 |  |
|  |  | L0L6K1 | 208 | 7 |  | 0.034 |  | 13.462 |  |
|  |  | L0L8B6 | 199 | 4 |  | 0.020 |  | 12.563 |  |
|  |  | Q9XGE9 | 366 | 3 |  | 0.008197 |  | 12.842 |  |
|  |  | Q9XGF0 | 369 | 3 |  | 0.00813 |  | 15.176 |  |
