# Supplementary material for: Mapping Coeliac Toxic Motifs in the Prolamin Seed Storage Proteins of Barley, Rye, and Oats Using a Curated Sequence Database
Source: Front Nutr. 2020 Jul 17;7:87. doi: 10.3389/fnut.2020.00087 (PMC7379453; doi:10.3389/fnut.2020.00087)
Supplement: Supplementary file 6 [file Table_6.DOCX]

**Supplementary Table S6.** **High molecular weight glutenin subunits identified in *T. aestivum* cvs. Chinese Spring and Hereward when searching mass spectrometry data against different GluPro databases.**  Mass spectrometry data were searched against GluPro v 1.0, v 1.1, v 1.2 and reviewed wheat prolamins in UniProt. The UniProt accession number, HMW glutenin subunit type, the number of peptides and unique peptides assigned to that protein, the unique peptide sequence and modifications on that peptide, alongside peptide score, sequence coverage and protein score. Bold and underlined residues indicate sites of modification.

| **Database** | **Cultivar** | **Accession number** | **Subunit type** | **Peptides (Unique peptides)** | **Unique peptide** | **Modifications** | **Peptide score** | **Sequence coverage (%)** | **Protein score** |
| --- | --- | --- | --- | --- | --- | --- | --- | --- | --- |
| UniProt reviewed  *T. aestivum* prolamins  N=43 | CS | P02861 | Ax | 2 (1) | YPTSPQQSGQGQQGY |  | 5.041 | 26.07 | 11.08 |
|  |  | P08489 | Dx2 | 44 (7) | **Q**QSGQGQHGY | [N-term] Pyroglutamic acid | 5.765 | 50.82 | 424.1 |
|  |  |  |  |  | **Q**QPEQGQPGY | [N-term] Pyroglutamic acid | 5.1443 |  |  |
|  |  |  |  |  | QQSGQGQHGY |  | 5.3744 |  |  |
|  |  |  |  |  | QQPEQGQPGYYPTSPQQPGQL |  | 7.1847 |  |  |
|  |  |  |  |  | AQGQQGQQPAQVQQGQQPAQGQQGQQL |  | 6.9919 |  |  |
|  |  |  |  |  | YPTSPQQPGQL |  | 6.6676 |  |  |
|  |  |  |  |  | YPGQASPQRPGQGQQPGQGQQSGQGQQGY |  | 7.0264 |  |  |
|  |  | P10388 | Dx5 | 40 (1) | **Q**QPEQGQPRYYPTSPQQSGQL | [N-term] Pyroglutamic acid | 5.2351 | 29.7 | 304.93 |
|  |  | P10387 | Dy10 | 18 (3) | YPTSLQQPGQGQQGY |  | 5.4051 | 24.25 | 150.34 |
|  |  |  |  |  | YPGVTS**P**RQGSY | [7] Hydroxyproline | 5.3587 |  |  |
|  |  |  |  |  | QQPGQGQQGHY |  | 6.4139 |  |  |
|  |  | P08488 | Dy12 | 20 (5) | Y**P**TSLQQPGQGQQIGKGK**Q**GYYPTSL | [2] Hydroxyproline\|[19] Deamidation Q | 5.4093 | 38.46 | 200.36 |
|  |  |  |  |  | GQGQQIGQVQQPGQGQQGY |  | 6.6831 |  |  |
|  |  |  |  |  | QQPGQQGHY**P**TSL | [10] Hydroxyproline | 5.5749 |  |  |
|  |  |  |  |  | QQPGQGQQIGQGQQGY |  | 6.8415 |  |  |
|  |  |  |  |  | QQPGQQGHYPTSL |  | 6.5902 |  |  |
|  | Hereward | P02861 | Ax | 2 (1) | YPTSPQQSGQGQQGY |  | 5.4418 | 52.48 | 5.42 |
|  |  | P08489 | Dx2 | 82 (14) | QQSGQGQHGY |  | 5.3439 | 78.76 | 499.03 |
|  |  |  |  |  | **Q**QPEQGQPGY | [N-term] Pyroglutamic acid | 5.0752 |  |  |
|  |  |  |  |  | YPGQASPQRPGQGQQPGQGQQSGQGQQGY |  | 5.0764 |  |  |
|  |  |  |  |  | YPTSPLQSGQGQPGYY |  | 5.0958 |  |  |
|  |  |  |  |  | AQGQQGQQPAQVQQGQQ**P**AQGQQGQQL | [18] Hydroxyproline | 5.1088 |  |  |
|  |  |  |  |  | YPTSPQQSGQGQQLGQW |  | 5.3065 |  |  |
|  |  |  |  |  | YPGQASPQRPGQGQQPGQGQQSGQGQQGYYPTSPQQPGQW |  | 5.3186 |  |  |
|  |  |  |  |  | E**QQ**IVVPKGGSFYPGETTPPQQL | [2] Deamidation Q\|[3] Deamidation Q | 5.5837 |  |  |
|  |  |  |  |  | LQPGQGQQGYYPTSPQQSGQGQQL |  | 5.6207 |  |  |
|  |  |  |  |  | GQGQQGQQPGQGQQPAQGQQGQQPGQGQQGQQPGQGQQPGQGQPW |  | 5.7286 |  |  |
|  |  |  |  |  | QQSGQGQHGYYPTSPQLSGQGQRPGQW |  | 5.7785 |  |  |
|  |  |  |  |  | AQGQQGQQPAQVQQGQQPAQGQQGQQL |  | 6.5839 |  |  |
|  |  |  |  |  | QQPEQGQPGYYPTSPQQPGQL |  | 6.6342 |  |  |
|  |  |  |  |  | YPTSPQQPGQL |  | 6.8449 |  |  |
|  |  | P10388 | Dx5 | 82 (11) | QQPEQGQPRYYPTSPQQSGQL |  | 5.159 | 53.24 | 442.85 |
|  |  |  |  |  | QQSGQGQHWYYPTSPQL |  | 5.1775 |  |  |
|  |  |  |  |  | YPTSPQQSGQL |  | 5.1835 |  |  |
|  |  |  |  |  | KRYYPSVT**C**PQQVSY | [9] Carbamidomethyl C | 5.2174 |  |  |
|  |  |  |  |  | YPTSPQESGQGQQPGQWQQPGQGQPGY |  | 5.2509 |  |  |
|  |  |  |  |  | **Q**PGQGQQGYYPTSPQ**QP**GQGQQLGQW | [1] Deamidation Q\|[16] Deamidation Q\|[17] Hydroxyproline | 5.2694 |  |  |
|  |  |  |  |  | YPTSPQQSGQGQPGYY |  | 5.3562 |  |  |
|  |  |  |  |  | Q**QP**EQGQPRYYPTSPQQSGQL | [2] Deamidation Q\|[3] Hydroxyproline | 5.3627 |  |  |
|  |  |  |  |  | **Q**QPAQGQQPG**Q**GQQGQQPGQGQQGQQPG**Q**GQQPGQGQPGY | [1] Deamidation Q\|[11] Deamidation Q\|[29] Deamidation Q | 5.4126 |  |  |
|  |  |  |  |  | **Q**QPEQGQPRYYPTSPQQSGQL | [N-term] Pyroglutamic acid | 5.547 |  |  |
|  |  |  |  |  | QQPEQGQPRYYPTSPQQSGQL |  | 5.159 |  |  |
|  |  |  |  |  | YYPTSPQL |  | 5.5919 |  |  |
|  |  | P10387 | Dy10 | 48 (11) | YPGVTSPRQGSY |  | 5.0511 | 53.09 | 304.67 |
|  |  |  |  |  | YPGVTS**P**RQGSY | [7] Hydroxyproline | 5.1574 |  |  |
|  |  |  |  |  | YY**P**TSLQQPGQGQQIGKGQQGY | [3] Hydroxyproline | 5.2454 |  |  |
|  |  |  |  |  | GQGQQPRQWQQSGQGQQGHY**P**TSL | [21] Hydroxyproline | 5.2834 |  |  |
|  |  |  |  |  | QQPGQGQQGHYPTSL |  | 5.3335 |  |  |
|  |  |  |  |  | YPTSLQQPGQGQQGY |  | 5.417 |  |  |
|  |  |  |  |  | QQPGQGQQGY |  | 5.4613 |  |  |
|  |  |  |  |  | QQPGQGQQIGKGQQGYYPTSL |  | 5.4622 |  |  |
|  |  |  |  |  | GQGQQPRQW |  | 5.6642 |  |  |
|  |  |  |  |  | **Q**QPGQGQQGYYPTSL | [N-term] Pyroglutamic acid | 5.8689 |  |  |
|  |  |  |  |  | WGTSSQTVQGYY**P**GVTS**P**RQGSY | [13] Hydroxyproline\|[18] Hydroxyproline | 5.9045 |  |  |
|  |  | P08488 | Dy12 | 44(8) | GQGQQPGQWQQSGQGQQGHYPTSL |  | 5.1693 | 58.01 | 290.11 |
|  |  |  |  |  | GQGQQPGQW |  | 5.2197 |  |  |
|  |  |  |  |  | YPSVTSPRQGSY |  | 5.378 |  |  |
|  |  |  |  |  | YPTSPQQLGQGQQPGQW |  | 6.3664 |  |  |
|  |  |  |  |  | GQGQQIGQVQQPGQGQQGYYPTSL |  | 6.3976 |  |  |
|  |  |  |  |  | QQPGQQGHYPTSL |  | 6.8539 |  |  |
|  |  |  |  |  | GQGQQIGQVQQPGQGQQGY |  | 6.9839 |  |  |
|  |  |  |  |  | QQPGQGQQIGQGQQGY |  | 7.1074 |  |  |
| GluPro v 1  N=630 | CS | Q41553 | Ax2 | 11 (1) | QPEQL |  | 5.0644 | 19.08 | 69.83 |
|  |  | G4Y3Y2 | Bx7.3 | 19 (1) | EQQPVVPSTAGSF |  | 5.9846 | 39.49 | 139.83 |
|  | Hereward | A0MZ38 | Ax | 10 (1) | LQ**P**GQWL | [3] Hydroxyproline | 5.3161 | 22.21 | 65.73 |
|  |  | Q6UKZ5 | Bx14 | 12 (3) | YPTSPQQSGQGQQSGQAQQGY |  | 5.1696 | 35.58 | 80.36 |
|  |  |  |  |  | YPISPQQSGQGQQTGQGQQGY |  | 5.2285 |  |  |
|  |  |  |  |  | QQVVDQQL |  | 5.4843 |  |  |
|  |  | Q52JL2 | By | 31 (2) | Y**P**TSPQQSGQGQQ**P**GQSQQPGQGQQGYYSGSL | [2] Hydroxyproline\|[14] Hydroxyproline | 5.0424 | 45.63 | 197.64 |
|  |  |  |  |  | Y**P**TSPQQSGQGQQPGQSQQPGQGQQGYYSGSL | [2] Hydroxyproline | 5.3687 |  |  |
|  |  | G3FLC7 | Dx2/3 | 48 (1) | YPTSPLQSGQG**Q**SGYYPTS**P**QQSGQGQQ**P**GQL | [12] Deamidation Q\|[20] Hydroxyproline\|[29] Hydroxyproline | 6.0494 | 57.36 | 315.01 |
| GluPro v 1.1  N=685 | CS | None identified | None identified | None identified | None identified | None identified | None identified | None identified | None identified |
|  | Hereward | Q6UKZ5 | Bx14 | 13 (3) | YPTSPQQSGQGQQPGQRQSGY |  | 5.3056 | 31.9 | 87.28 |
|  |  |  |  |  | QQVVDQQL |  | 5.486 |  |  |
|  |  |  |  |  | YPTSSQQSGQGQQPGQGQ**P**GY | [19] Hydroxyproline | 5.7083 |  |  |
|  |  | Q52JL2 | By | 31 (3) | TSQQQPGQGQQRHYPASL |  | 5.017 | 47.83 | 196.86 |
|  |  |  |  |  | Y**P**TSPQQSGQGQQPGQSQQPGQGQQGYYSGSL | [2] Hydroxyproline | 5.3195 |  |  |
|  |  |  |  |  | YSGSLQQPGQGL |  | 5.3286 |  |  |
|  |  | G3FLC7 | Dx2/3 | 47 (1) | YPTSPLQSGQG**Q**SGYYPTS**P**QQSGQGQQ**P**GQL | [12] Deamidation Q\|[20] Hydroxyproline\|[29] Hydroxyproline | 5.975 | 56.16 | 303.04 |
|  |  | Q52JL3 | Dy12 | 22 (1) | YPTSPQQPGQGQQGHHPASL |  | 5.2188 | 46.81 | 175.38 |
| GluPro v 1.2  N=699 | CS | None identified | None identified | None identified | None identified | None identified | None identified | None identified | None identified |
|  | Hereward | Q6UKZ5 | Bx14 | 15 (5) | QQPRQGQQSGQGQPGY |  | 5.0278 | 46.18 | 97.57 |
|  |  |  |  |  | YPTSPQQSGQGQQSGQAQQGY |  | 5.1572 |  |  |
|  |  |  |  |  | YPTSPQQSGQGQQPGQRQSGY |  | 5.3061 |  |  |
|  |  |  |  |  | QQVVDQQL |  | 5.4886 |  |  |
|  |  |  |  |  | YPTSSQQSGQGQQPGQGQ**P**GY | [19] Hydroxyproline | 5.712 |  |  |
|  |  | Q52JL2 | By | 28 (2) | YSGSLQQPGQGL |  | 5.3283 | 46.72 | 182.53 |
|  |  |  |  |  | Y**P**TSPQQSGQGQQPGQSQQPGQGQQGYYSGSL | [2] Hydroxyproline | 5.3288 |  |  |
|  |  | G3FLC7 | Dx2/3 | 46 (1) | YPTSPLQSGQG**Q**SGYYPTS**P**QQSGQGQQ**P**GQL | [12] Deamidation Q\|[20] Hydroxyproline\|[29] Hydroxyproline | 5.9764 | 55.99 | 293.4 |
|  |  | Q52JL3 | Dy12 | 21 (1) | YPTSPQQPGQGQQGHHPASL |  | 5.2218 | 46.18 | 170.81 |
