# Supplementary figures and images for: Mapping Coeliac Toxic Motifs in the Prolamin Seed Storage Proteins of Barley, Rye, and Oats Using a Curated Sequence Database
Source: Front Nutr. 2020 Jul 17;7:87. doi: 10.3389/fnut.2020.00087 (PMC7379453; doi:10.3389/fnut.2020.00087)

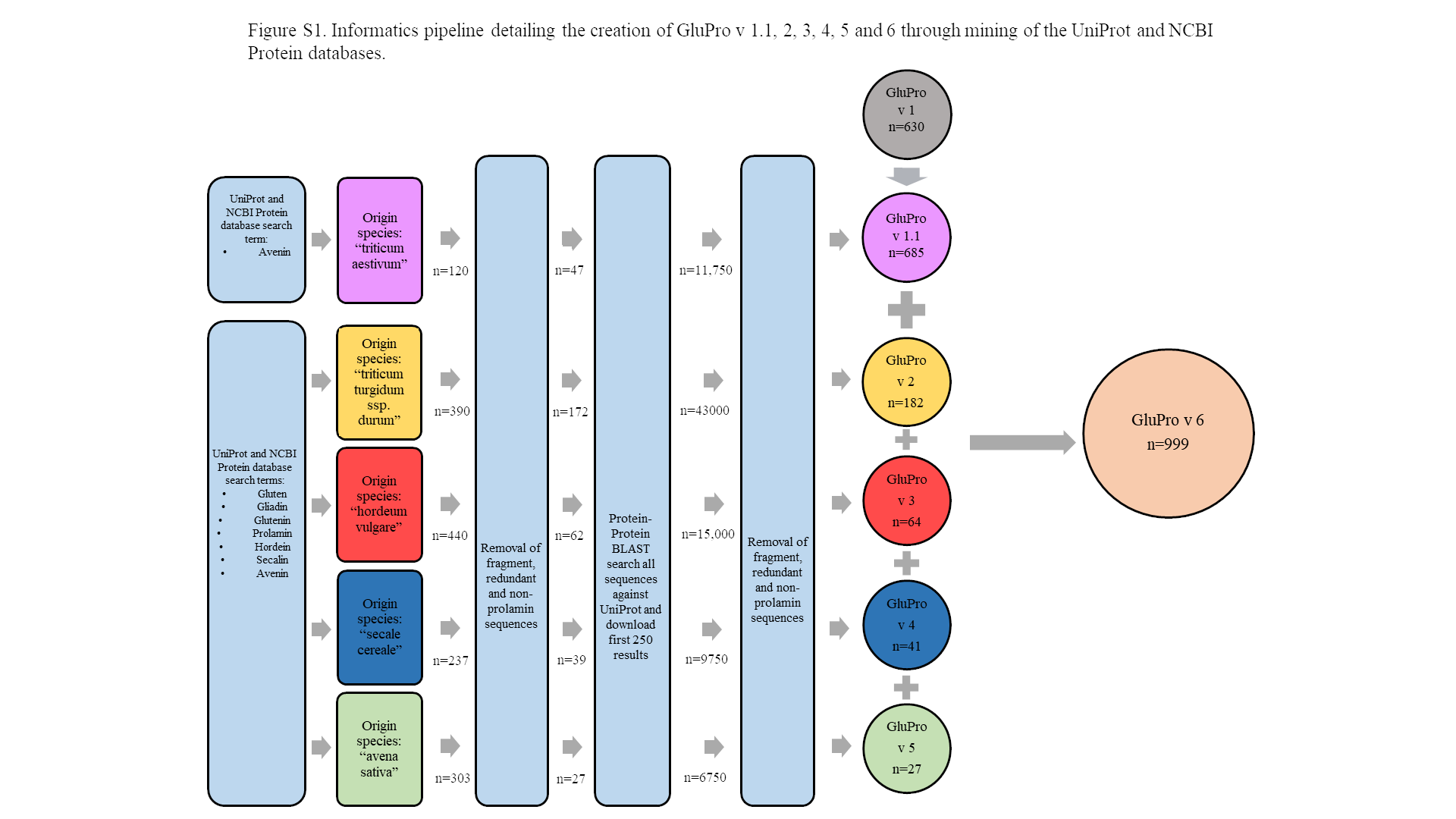

Supplement: Supplementary file 7 [file Image_1.TIF]

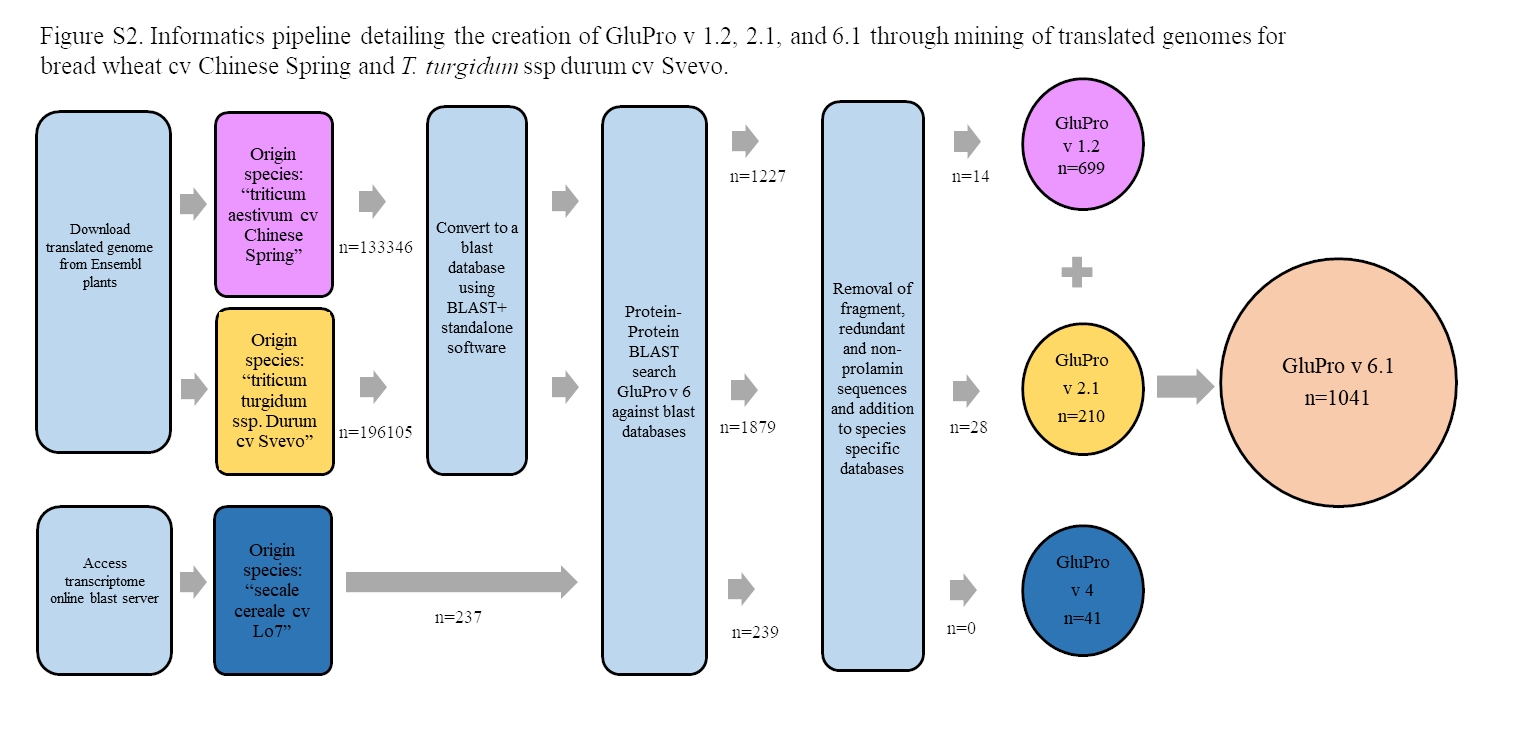

Supplement: Supplementary file 8 [file Image_2.TIF]

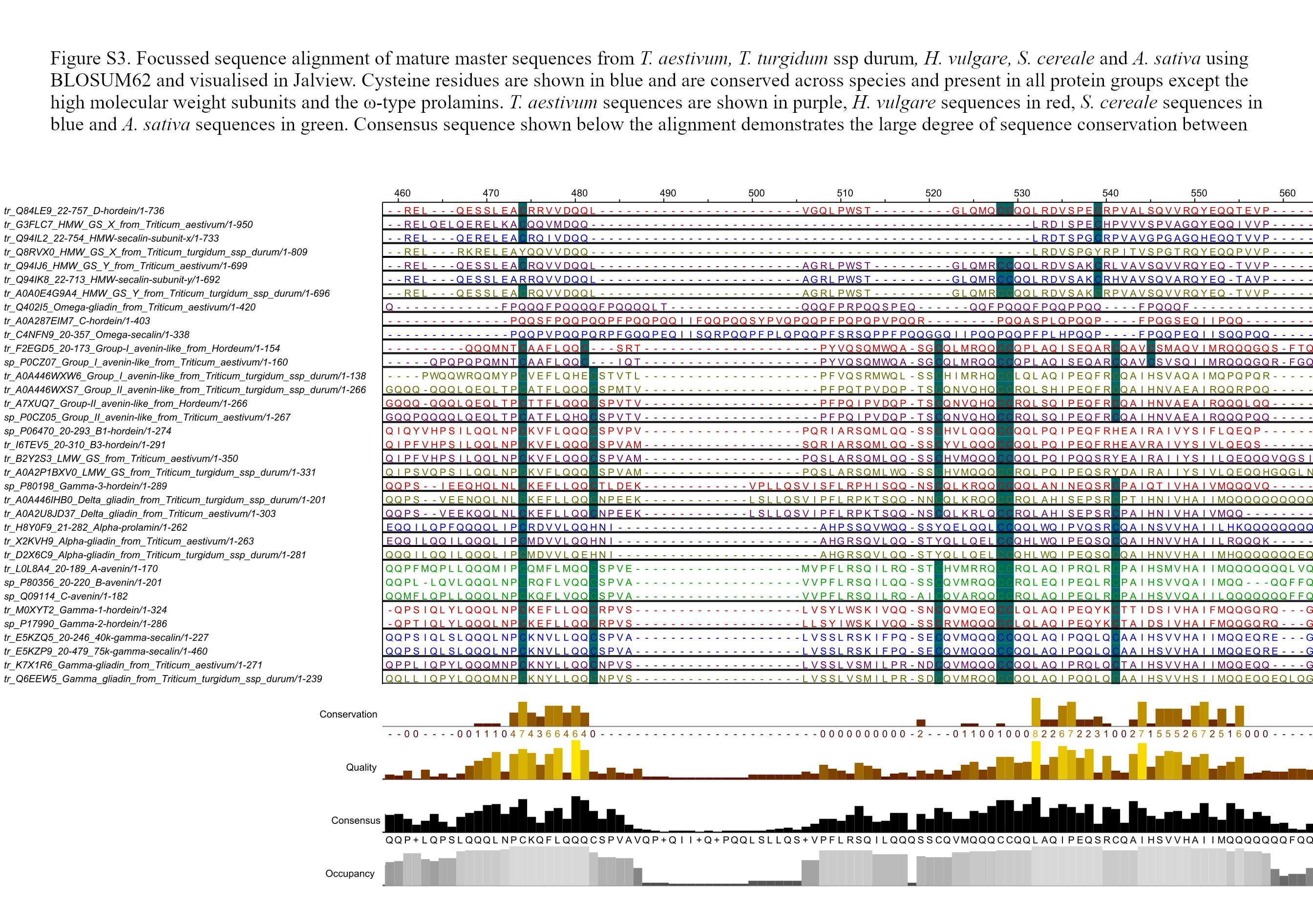

Supplement: Supplementary file 9 [file Image_3.TIF]

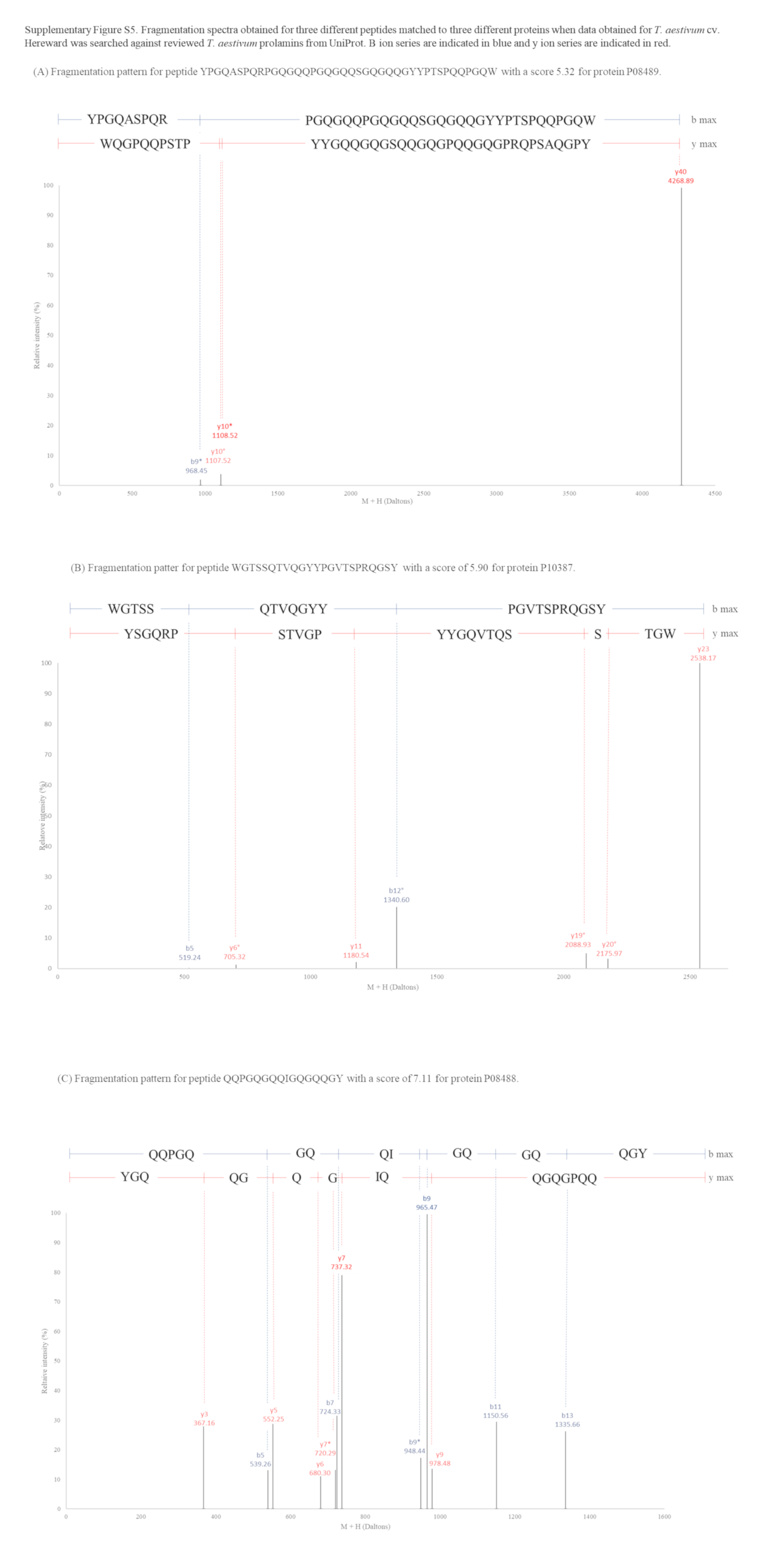

Supplement: Supplementary file 11 [file Image_5.tif]
