# Supplementary material for: Mapping Coeliac Toxic Motifs in the Prolamin Seed Storage Proteins of Barley, Rye, and Oats Using a Curated Sequence Database
Source: Front Nutr. 2020 Jul 17;7:87. doi: 10.3389/fnut.2020.00087 (PMC7379453; doi:10.3389/fnut.2020.00087)

Supplementary Figure S7. Frequency distribution of q values of identified proteins when searching *T. aestivum* cv Chinese Spring (A) and cv Hereward (B) mass spectrometry data against different curated sequence databases.

(A)

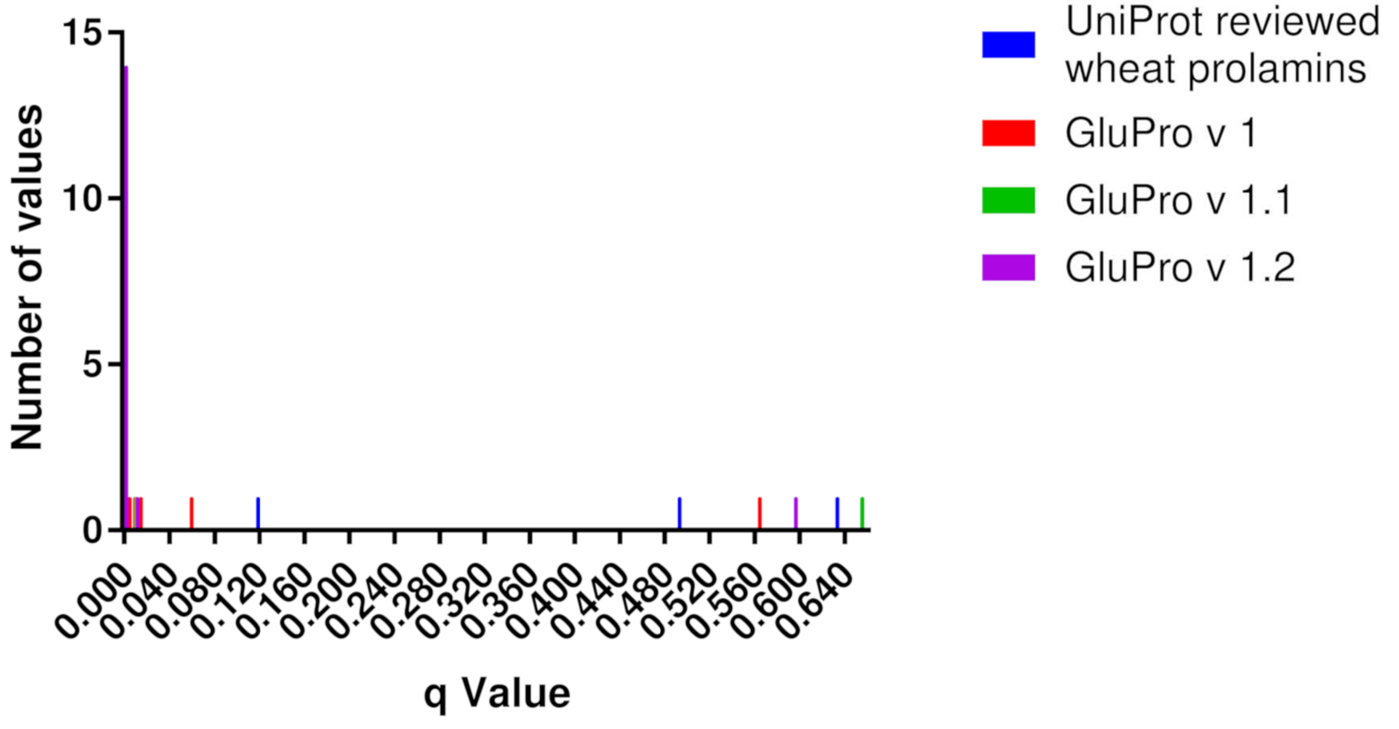

(B)

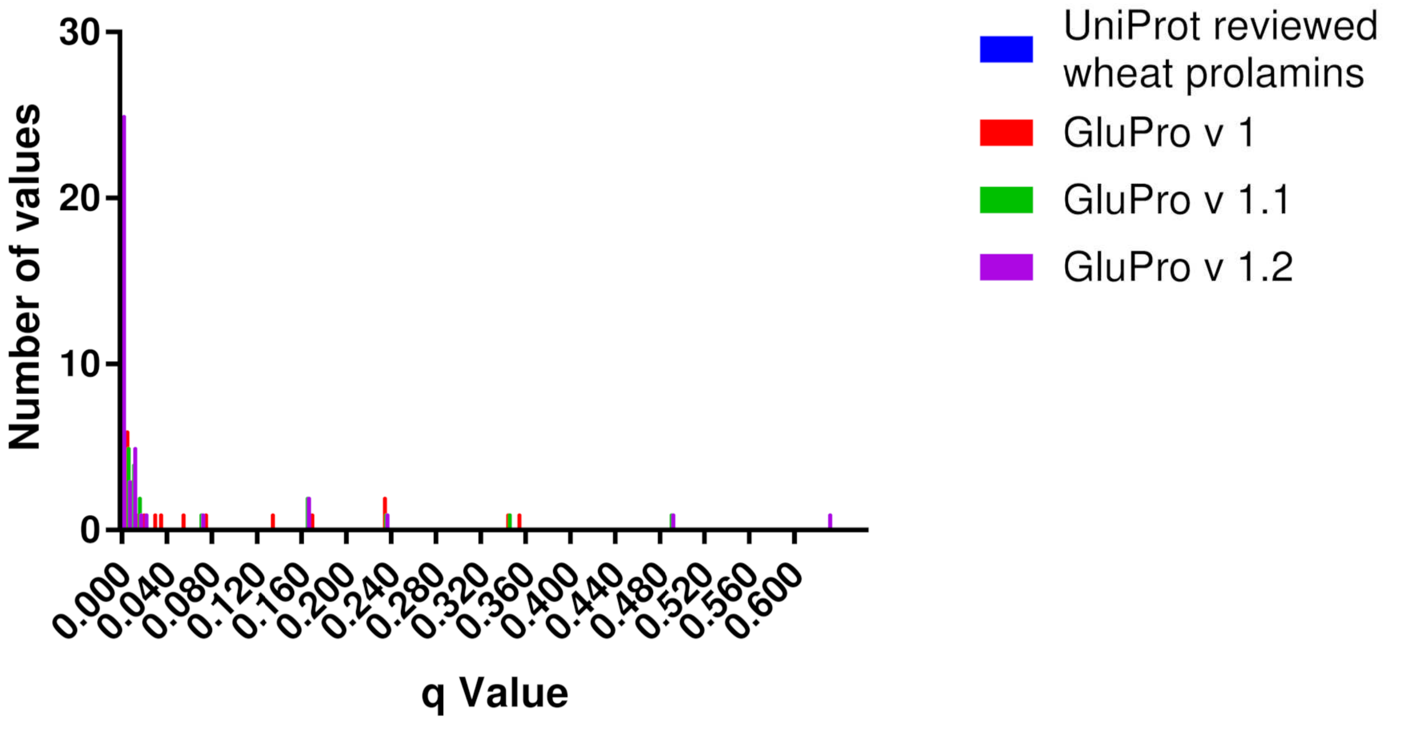

Supplement: Supplementary file 13 [file Image_7.pdf]
